# PPy‐Coated Wire Actuators for the Micromechanostimulation of Cells: Fabrication and Characterization

**DOI:** 10.1002/smsc.202500639

**Published:** 2026-03-13

**Authors:** Amaia B. Ortega‐Santos, Satoru Hayano, Emilio Satoshi Hara, Jose G. Martínez, Hiroshi Kamioka, Edwin W. H. Jager

**Affiliations:** ^1^ Sensor and Actuator Systems Department of Physics Chemistry and Biology (IFM) Linköping University Linköping Sweden; ^2^ Department of Orthodontics Okayama University Hospital Okayama Japan; ^3^ Advanced Research Center for Oral and Craniofacial Sciences Dental School Okayama University Graduate School of Medicine Dentistry and Pharmaceutical Sciences Okayama Japan; ^4^ Department of Orthodontics Okayama University Graduate School of Medicine Dentistry and Pharmaceutical Sciences Okayama Japan

**Keywords:** conducting polymers, mechanotransduction, osteoblasts, polypyrrole, RNA sequencing, soft‐microactuators

## Abstract

Cellular mechanotransduction signals play a crucial role in physiological and pathological conditions, including skeletal disorders. Although various systems exist to mechanically stimulate cultured cells, most are constrained by incubator incompatibility, limited physiological relevance, nonuniform stimulation, or complexity. The objective of this article is to develop and validate a compact, incubator‐compatible tool capable of delivering localized and physiologically relevant mechanical stimulation to small cell populations. Here, we introduce a polypyrrole‐based wire‐shaped microactuator designed to induce localized mechanical stress to adjacent cells. These wire‐shaped microactuators are biocompatible, easy‐to‐use, and compact for use within standard in vitro cell culture systems. Using a noncontact optical method, we characterize the actuation of polypyrrole‐coated wires in an aqueous NaDBS electrolyte, showing radial expansion of 1.5–8 µm depending on the deposited polypyrrole film thickness, comparable to cellular dimensions. Next, the actuation is confirmed to be robust and stable to use in cell culture media at physiological temperature. To evaluate biological relevance, osteoblastic KUSA‐A1 cells are mechanically stimulated inside the incubator and transcriptomic changes are assessed. Mechanical stimulation resulted in upregulation of genes previously associated with mechanotransduction, including Fos and Fosb. Additionally, several uncharacterized long noncoding RNAs are differentially expressed, suggesting potential novel players in the mechanotransduction pathway.

## Graphical abstract

“Ouch!” That's what we say when someone pushes us. Cells do the same. They sense mechanical cues and trigger downstream signals shaping physiological and pathological responses. While the basics of mechanotransduction are known, many pathways remain unexplored. We present a user‐friendly tool that mimics native mechanical cues, enabling new insights into RNA changes driven by cellular force sensing.

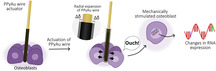

## 1 Introduction

Cells are constantly exposed to mechanical cues in their native environments, from fluid shear and compression to tension and matrix stiffness. Cells actively sense and convert these cues into biochemical signals through a process known as mechanotransduction. Sensing occurs via transmembrane proteins such as integrins, which connect the extracellular matrix (ECM) with the cytoskeleton [1]; stretch‐activated ion channels that open in response to membrane tensions or stress [2]; cadherins, which are cell–cell junctions that sense mechanical forces coming from neighboring cells [3]; and specialized surface processes, i.e., morphological protrusions or organelles that function as mechanosensors, such as primary cilia or microvilli [4]. Once the mechanical forces have been sensed, the cytoskeleton transmits the forces via the cytoskeletal network to deeper structures of the cell, like the nucleus [5, 6]. This mechanical input can change the nuclear architecture and activate mechanosensitive transcription factors, leading to changes in RNA expression [7, 8]. In particular, these transcriptional shifts initiate cascades of downstream mechanoadaptation effects, including cytoskeletal reorganization [9, 10, 11], focal adhesion formation and size modulation [1, 12], calcium (Ca^2+^) signaling [9, 13], activation of ion channels [14, 15], and changes in cellular function, e.g., cell migration, proliferation, differentiation, and apoptosis [16].

While the overall framework on how cells sense mechanical forces is well‐established, many underlying pathways, molecular interactions, and microenvironment‐dependent responses are still not fully understood. There are cell‐type‐specific differences, not only due to their different cell type function but because of their microenvironment differences, e.g., endothelial cells form a flat 2D layer lining blood vessels, while myocytes are embedded in a 3D ECM matrix. Consequently, cells are also exposed to distinct mechanical forces (e.g., shear, pressure, stiffness, tension) which are sensed and interpreted differently [17, 18]. These mechanical inputs give rise to localized forces at the single‐cell level as well as dynamic and heterogeneous mechanical microenvironments, and cellular responses often depend on the combined action of multiple mechanical cues. Moreover, how cells integrate short‐term versus long‐term mechanical stimulation, as well as cues delivered at different frequencies or strain amplitudes, to produce distinct biological outcomes is still not fully understood. Importantly, the boundary between mechanically regulated processes that support normal cellular function and those that contribute to disease is often subtle and context dependent [19, 20].

Addressing these open questions in cellular mechanotransduction is essential for revealing fundamental principles of cell biology and also for developing effective strategies to address mechanically driven diseases, such as cancer, osteoarthritis, or thrombosis [20, 21]. In vitro mechanostimulation models are needed to systematically study these processes, and the tools used must meet several key requirements. They should be compatible with standard cell culture conditions and adaptable to different culture formats, from surface coatings to 3D matrices. The systems must provide well‐controlled mechanical stimulation with defined parameters through simple operation, ideally within the well plate. Localized stimulation is also desirable which will allow distinguishing directly stimulated cells from neighboring ones and studying cell–cell communication. Finally, such tools should be extendable to more complex tissue models and potentially in vivo settings to enable biological comparison.

Despite major advances, current mechanostimulation methods do not simultaneously meet all these requirements and face some limitations, e.g., non‐uniform stimulation, disparities from physiological conditions, and high costs or time requirements. Existing techniques can be categorized based on the scale of stimulation, each with distinct advantages and drawbacks. For the stimulation of a large population of cells, methods such as cell culture deformation and flow channels for shear forces are commonly employed. Commercial systems like the FlexCell FX‐4000 C use air pressure to deform tissue samples or cell cultures on the flexible bottom of custom culture dishes. However, this system exhibits nonuniform stimulation and cannot be operated inside an incubator. The latter limits the time of the mechanical stimulation and lacks equivalent physiological conditions [22, 23]. Flow channels, which are microfluidic systems that expose cells to a constant flow of a fluid, provide controlled shear forces but are restricted to this specific type of mechanical stimuli [24, 25, 26, 27]. For a few hundred cells, different approaches typically involve the use of serial or parallel configurations of magnetic or electric fields [28], sometimes combined with microfluidic devices [29]. Conducting polymers (CPs) have also been utilized for their versatility and possibility to be miniaturized [13, 30]. However, the authors reported the inability of the system to cycle the expansion and contraction of the PPy, limiting the system to a single expansion cycle rather than a sequence of pulses. For single cells, a wide array of methods is available, including atomic force microscopy (AFM) [31, 32, 33, 34, 35], electric fields [36, 37, 38, 39], magnetic fields [12, 40, 41, 42, 43], micropipette aspiration [44], or optical traps [45, 46, 47]. These approaches enable precise manipulation and analysis of individual cells but are technically demanding and deviate from physiological conditions, as most cells in vivo are adhered to an ECM and neighboring cells. This adherence is crucial for maintaining normal cell behavior. Additionally, the limited capacity of single‐cell methods makes statistical analysis challenging.

Soft actuators are composed of compliant materials that enable flexible, adaptable motion, and are a promising alternative for mechanostimulation. These actuators can be powered via electrical fields or currents, thermal energy, magnetic fields, light, and (bio)chemical stimuli, resulting in diverse strain outputs [48]. Among them, CP‐based (micro‐)actuators are electrochemically driven, with high control over actuation, fast response, and programmability. These actuators are versatile, easy to use, and operate at very low potentials (less than 1 V), enabling actuation on the micrometer scale with linear strain values of up to 20%–26% and perpendicular strains of 30%–51% [49, 50, 51, 52]. CPs are biocompatible, ensuring good cell viability when interfaced with living cells [53, 54, 55, 56]. CP‐based actuators can be fabricated using different CPs, such as Poly(3,4‐ethylenedioxythiophene) (PEDOT) and Polyaniline (PANI). In particular, polypyrrole (PPy) has shown great actuation properties and has already been successfully employed for epithelial cell mechanostimulation [13]. These properties make PPy‐based actuators ideal tools for investigating the cellular response to mechanical stimuli in physiological environments.

The actuation characterization of CP actuators is crucial for optimizing their performance. The underlying mechanism is based on the mass exchange, i.e., ions and solvent, from and to the electrolyte upon electrical stimuli of the material [57, 58]. While most studies focus on the actuation of CP actuators in standard electrolytes, e.g., aqueous NaDBS solutions, it has not yet been systematically investigated in more complex environments, like cell culture media [53, 59]. Techniques such as AFM are used to assess out‐of‐plane volume changes, while isotonic measurements enable the direct evaluation of length variations [60, 61]. We have previously reported a noncontact optical method that monitors the shadow cast between an emitter and detector [52], which for tubular samples corresponds to the diameter of the objects.

**FIGURE 1:**
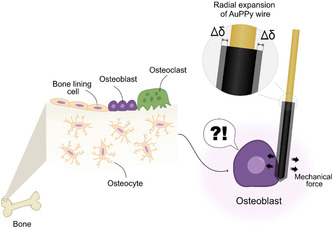
Mechanostimulation of cell via PPyAu wire actuators, concept figure.

Here, we hypothesize that biocompatible PPy microactuators could generate mechanical displacements of sufficient magnitude to induce cellular mechanostimulation while operating under physiologically relevant cell culture conditions. PPy could be deposited on a conductive wire that could be easily inserted into standard well plates and could be operated inside the incubator. To test this hypothesis, we developed a novel PPy‐based tool in the form of a PPy coated straight gold wire (named PPyAu wire actuator) designed for cell mechanostimulation. The PPyAu wire actuator is placed inside standard well plates to generate localized microactuation adjacent to the cells, thereby inducing mechanical stimulation. Figure 1 shows the principle of cell mechanostimulation using the PPyAu wire actuator. This article is structured into two parts: the main focus is to provide an electrochemical and mechanical characterization of the PPy deposition and actuation using a previously developed noncontact optical measurement technique. We investigated how PPy film thickness influenced actuation behavior in NaDBS aqueous solution and demonstrated that the generated strain could be tuned by both the deposited PPy thickness and the duration of the applied electrical pulse. Actuation performance was evaluated using standard two‐ and three‐electrode electrochemical configurations to assess adaptability to different well plate sizes and formats, including small‐volume culture systems. The actuator performance was subsequently validated in cell culture media at both room and physiological temperature. In addition, long‐term cyclic actuation (up to 3 h) and extended stimulation pulses (up to 30 min) in cell media were evaluated to assess actuator stability, durability, and flexibility across different stimulation protocols.

In the last part, we complemented this work by presenting a proof‐of‐concept experiment, demonstrating the potential of the PPyAu wire actuator as a mechanostimulation tool by penetrating KUSA‐A1 osteoblastic cells seeded on transwell membrane inserts in a standard 24‐well plate, and stimulating these cells inside an incubator. Cells located adjacent to the PPyAu wire actuator were collected, and mechanically induced transcriptomic changes were analyzed by RNA sequencing. This work establishes the in vitro applicability of the proposed approach and demonstrates that key requirements for physiologically relevant mechanostimulation are met.

This study highlights the potential of CP‐based (micro‐)actuators for cell mechanostimulation research and for developing novel tools for fundamental cell studies and tissue engineering applications.

## 2 Methods

### 2.1 Materials

Ag wires (99.99%, diameter of 800 μm, CAS nr. 7440‐22−4) and Au wires (99.95%, diameter of 500 μm; CAS nr. 7440‐57−5) were purchased from GoodFellow Inc (Cambridge Ltd, UK) and cut to the desired length before use. Pyrrole (98%, Sigma–Aldrich, Darmstadt, Germany; CAS nr. 109‐97−7) was distilled under vacuum and stored at −20°C before use. Sodium dodecylbenzenesulfonate (NaDBS; CAS nr. 25 155‐30−0) was purchased from Sigma–Aldrich, stored at room temperature and used as received. Minimum Essential Medium Eagle M4526 (MEM) cell media was purchased from Sigma–Aldrich and stored at 4°C. Hydrogen peroxide (25% H_2_O_2_, stored at 4 ^o^C; CAS nr. 7722‐84−1) and ammonia (30% NH_4_, stored at RT; CAS nr. 1336‐21−6) were purchased from VWR and used as received. L‐glutamine– and phenol red–containing α‐Minimum Essential Medium (α‐MEM; CAS nr. 135–15 175) was purchased from FUJIFILM Wako Pure Chemical Corporation (Osaka, Japan). Fetal bovine serum (FBS, 10%; CAS nr. 175 012) was obtained from NICHIREI BIOSCIENCES INC. (Tokyo, Japan). Penicillin–streptomycin solution (P/S, 1%; CAS nr. 15 140–122) was purchased from Gibco (Grand Island, NY, USA). Atelocollagen permeable membrane culture inserts (AteloCell; CAS nr. CM‐24) were obtained from KOKEN Co., Ltd. (Tokyo, Japan). Standard 24‐well tissue culture plates (Costar; CAS nr. 3738) were purchased from Corning Inc. (Grand Island, NY, USA). Laboratory‐grade silicone was obtained from Shofu (Kyoto, Japan). RNA extraction was performed using the RNeasy Mini Kit (Qiagen, Hilden, Germany). Last, 5 mm diameter (area ≈ 20 mm^2^) biopsy punch was purchased from KAI Industries Co., Ltd., (Gifu, Japan).

### 2.2 Fabrication and Characterization of PPy‐Coated Au Wires

#### 2.2.1 Noncontact Optical Measurements

**FIGURE 2:**
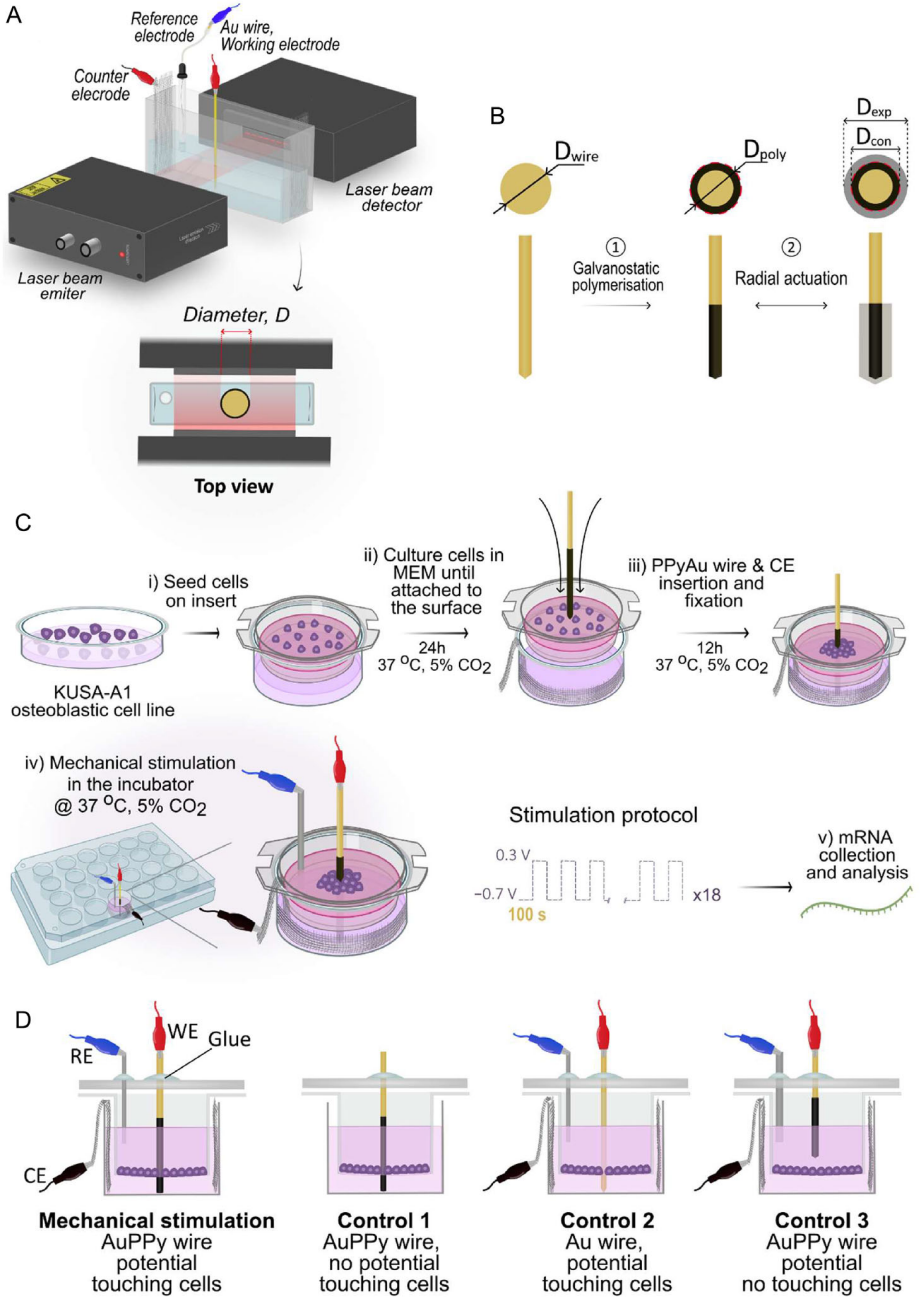
(A) Illustration of the combination of the laser scanner micrometer with the three‐electrode electrochemical cell and the top‐view of the laser scanner and PPy‐coated Au wire blocking the light and creating the shadow in the detector. (B) Process flow of the preparation of PPyAu wire actuator: before polymerization (bare Au wire), right after the polymerization (poly) which includes a PPy coating, and an expanded (exp) and contracted (con) polymer after the actuation of the polymer. Illustrations are not made to scale. (C) Cell culture and cell stimulation process flow. (D) Stimulation and controls illustrations.

For the fabrication and the characterization of the actuation of PPyAu wires a three‐electrode electrochemical cell set up and laser scanner micrometer (LSM) for noncontact optical measurements were used synchronously (Figure 2).

The working principle of the LSM is illustrated in Figure 2A. The LSM is composed of two parts: the beam emitter and the beam detector (Mitutoyo LSM‐501H), and the controller (Mitutoyo LSM‐6100). The beam emitter scans a horizontal array of coherent, highly collimated, 640 nm red light that is detected by the beam detector. When the Au or PPyAu wire actuator is placed perpendicularly between the emitter and the detector, the PPyAu wire actuator partially blocks the light beam and a shadow, equal to the diameter of the wire, is created on the detector (Figure 2A, top view). By continuously monitoring changes in the shadow, real‐time diameter changes can be measured. Illustrations of the cross sections of the wire showing its diameter at different stages (noncoated, coated with PPy after polymerization and upon PPy actuation) are shown in Figure 2B. A diameter‐equivalent voltage output signal from the LSM controller is sent to the computer, which is combined with the electrochemical data provided by the potentiostat. The thickness of the electropolymerized PPy (Polymerization thickness), the radial thickness variation during the actuation (Expansion/Contraction), and the strain of the actuation of the PPyAu wire actuator (Strain) were calculated from the changes on the diameter with the following equations:

(1) $$\text{Polymerisation thickness }(\text{µ}m):{\text{δ}}_{\text{poly}}=\frac{D_{\text{poly}}-D_{\text{wire}}}{2}$$

(2) $$\text{Expans}\mathrm{ion}/\text{Contraction }(\text{µ}m):\text{Δ}\text{δ}=\frac{D_{\text{expanded}}-D_{\text{contracted}}}{2}$$

(3) $$\text{Strain }\left(\%\right)=\frac{\text{Δ}\text{δ}}{{\text{δ}}_{\text{poly}}}100$$

where poly refers to polymerization, *D*_wire_ to the uncoated wire diameter, and *D*_expanded_ and *D*_contracted_ to the swollen and shrunk diameter of PPy‐coated Au wire during actuation after the process has stabilized. *δ* represents thickness, and Δ*δ* increment of thickness [52].

#### 2.2.2 Electropolymerization and Electrochemical Measurements

Prior to the electropolymerization of PPy on the Au wires, the Au wires were cleaned in a 5:1:1 DI water (18.2 MOhm, Purelab Chorus Elga from Veolia Water Technologies, High Wycombe, Essex, UK), 30% H_2_O_2_, 25% NH_3_ solution at 100°C for 15 min and subsequently rinsed with DI water ten times. The Au wire was soldered to a banana connector (RS components, London, UK), and nonconductive heat shrink tubing was added to the upper part to ensure that the conductive length (*L* = 1.8 cm for the characterization and *L* = 1.5 cm for the stimulation of cells) of the wire remained constant during all the experiments. Then, the 500 μm diameter Au wire (working electrode, WE) was suspended and positioned in a rectangular glass cuvette (5.5 × 5.2 × 2.9 cm, Starna Scientific Ltd, Hainault, UK), which was placed between the emitter and detector of the LSM. Two stainless‐steel meshes, positioned parallel on two sides of the cuvette, outside the laser array range, were used as the CE. By positioning the Au wire in the middle of those two CEs, comparable working‐electrode−counter‐electrode distances were ensured, resulting in a symmetric electric field which promotes uniform and symmetric polymerization and actuation [52]. An Ag/AgCl (3 м NaCl) reference electrode (RE) (MF‐2052 model from BASi Research Products, West Lafayette, Indiana, USA) or a silver wire pseudo‐RE was also suspended, clamped with conducting crocodile clips, and positioned outside the laser array. Polypyrrole was galvanostatically synthesized at + 0.3 mA cm^−1^ (per cm of length of the Au wire immersed in solution during electropolymerization) from a 0.1 м pyrrole and 0.1 м NaDBS aqueous solution onto the Au wire (Figure 2B, step 1). The electropolymerization parameters were selected based on previously established and validated protocols reported in earlier work [52]. The deposition was performed in 15 mL of the forementioned solution for durations of 4, 8, 12, 20, 30, 40, and 60 min. The electroactivity of the PPyAu wire actuator was verified after polymerization by performing cyclic voltammetry (CV). This was conducted between −1.0 and + 0.5 V or between −1.0 and + 1.5 V (vs. Ag/AgCl, 3 M NaCl) at a scan rate of 10 mV s^−1^ for 15 cycles, both in 0.1 м NaDBS and in MEM, at room temperature or 37°C. The radial actuation of the PPyAu wire was assessed after CV by applying square‐wave potential pulses between −0.7 and + 0.3 V (vs. Ag/AgCl, 3 м NaCl). The pulses were applied for 150 s and 500 s in 0.1 м NaDBS and 150 s in MEM at each potential limit (Figure 2B, step 2). The control and measurement of the electrochemical parameters were conducted using a Metrohm AutoLab PGSTAT204 potentiostat together with the supplied Nova 2.1 software. Each experiment was repeated at least three times to ensure reproducibility.

#### 2.2.3 Elemental Component Analysis

For the elemental component analysis, samples were actuated with the same actuation protocol used for the characterization in both NaDBS and MEM and left either in the reduced or oxidized state by stopping the 150 s long square wave potential pulses at the end of either the reduction or oxidation potential respectively. Then, the samples were mounted onto an aluminum holder with carbon tape. Elemental components were evaluated using energy‐dispersive X‐ray spectroscopy (EDX, Oxford Instruments, UK) with Leo1550 Gemini Scanning Electron Microscope (Zeiss, Germany) and an electron acceleration voltage of 10 kV.

#### 2.2.4 Brightfield Imaging

Noncontact optical measurements using the LSM were additionally validated by live actuation of the PPyAu wires using a fluorescence microscope (x83, Evident, Tokyo, Japan) equipped with a 10x to 40x objective, under room temperature conditions. Time‐lapse images of the actuation of the PPyAu wire were acquired at 1 frame every 2 s over 6 full redox cycles (from −0.7 V to + 0.3 V, 150 s per step). Two experimental conditions were performed: (1) wires immersed in cell culture medium alone, and (2) actuation in the presence of KUSA‐A1 osteoblastic cell line seeded on the insert membrane, where the PPyAu wire was positioned in direct contact with the cells and penetrated the membrane. The PPyAu wire was connected to a three‐electrode system using PGSTAT204 potentiostat together with Nova 2.1 software. All images were acquired using cellSens software (Evident).

### 2.3 Mechanostimulation of Cells

#### 2.3.1 Cell Culture

KUSA osteoblastic cell line was kept in Minimum Essential Medium Alpha with L‐glutamine and phenol red with 10% fetal bovine serum and 1% penicillin–streptomycin. Cultures were incubated at 37°C in a humidified atmosphere containing 5% CO_2_.

For each experiment, the cells were cultured in atelocollagen permeable membrane culture inserts designed for 24‐well plates and cultured for 24 h to allow the cells to adhere to the membrane (Figure 2C). The atelocollagen membrane in the inserts mimics the compliant extracellular environment. For each culture, 500 μl of cell suspension containing 1.5 × 10^5^ cells (sufficient to achieve confluency) was added inside the insert, and an additional 500 μl of complete culture medium was added to the outer compartment to ensure proper hydration and nutrient exchange across the membrane. The surface area of each membrane was 64 mm^2^.

#### 2.3.2 Stimulation of KUSA‐A1 Osteoblastic Cell Line

Cell stimulation was conducted using a three‐electrode electrochemical setup adapted to a 24‐well plate. To achieve this, the standard stainless‐steel mesh was replaced with a thin, flexible stainless‐steel S/S woven wire mesh (Easipet), cut into a “T” shape. This sheet was positioned along the walls of the well plate, covering the inner surfaces while leaving a portion exposed outside the plate for connection to electrical leads (like shown in Figure 2C, steps iii to iv). Two 1 mm diameter holes were made in the lid of the well plate to allow vertical insertion of the Ag pseudo‐RE and the PPyAu actuator into the well, positioned near the side and center of the insert, respectively.

Prior to stimulation, PPyAu wire actuators with 20‐µm‐thick polypyrrole were freshly synthesized as described previously. The actuators were pre‐actuated in 0.1 м NaDBS by applying square wave potentials ranging from −0.7 to + 0.3 V for ten cycles, with each potential maintained for 150 s. The wires were then disinfected by immersion in 70% ethanol for 30 s and washed with MEM cell culture medium; this sequence was repeated twice.

After the cells were incubated on the inserts for 24 h to allow cell adhesion (Figure 2C, step i), the PPyAu wire actuators were vertically inserted through the lid, piercing the membrane and coming into direct contact with the cells (Figure 2C, step ii). The PPyAu wire actuators were secured in place with lab silicone (Shofu, Kyoto, Japan) to prevent unintentional mechanical stimulation from cable manipulation. The Ag pseudo‐RE was similarly inserted outside the cell area without contacting the cells and fixed with lab silicone. The three‐electrode setup was then connected to the potentiostat using thin cables and the cables were accessible from outside the incubator, enabling the incubator to remain closed during the mechanostimulation experiments. The cells were allowed to equilibrate under these conditions for at least 12 additional hours before mechanical stimulation (Figure 2C, step iii).

The stimulation of the cells was performed by applying 18 cycles of square wave potentials stimuli ranging from −0.7 to + 0.3 V, with 100 s at each potential limit (Figure 2C, step iv). Figure 2D illustrates the position of the PPyAu wire actuator during the stimulation (*Stimulation*) and controls inside the well plate. Three different experiments were designed as controls. For *Control 1*, a PPyAu wire actuator was inserted through the cell‐seeded membrane without applying any current but in contact with the cells to assess the effect of the material itself. Ionic current flow in cell culture media can modify the extracellular electric field experienced by cells, which in turn can influence the transmembrane potential and affect cellular functions [62]. Therefore, for *Control 2*, a noncoated Au wire was inserted, penetrating the cell membrane and placing it again in contact with the cells, and the same stimulation protocol was applied. For *Control 3*, a PPyAu wire actuator was inserted and placed just above the membrane, without contacting the cells, while the same protocol was applied too. For easier handling, each stimulation was performed in independent well plates. These plates were removed from the incubator immediately after stimulation for RNA extraction (Figure 2C, step v).

#### 2.3.3 RNA Extraction and Sequencing

RNA was extracted using the RNeasy Mini Kit according to the protocol supplied by the manufacturer. To isolate RNA specifically from the central region of the culture insert, a 5 mm diameter biopsy punch was used. By using this targeted collection approach, nearly one‐third of the total membrane area (64 mm^2^) was collected for gene expression analysis, reducing cross‐contamination of stimulated and nonstimulated cells, and providing accurate characterization of mechanosensitive responses. Extracted RNA samples were submitted to Azenta Life Sciences (South Plainfield, NJ, USA) for RNA sequencing analysis. Library preparation, high‐throughput sequencing, and initial bioinformatics processing were performed by the service provider using standardized protocols.

## 3 Results

### 3.1 Fabrication and Characterization of PPyAu Wires

#### 3.1.1 Electropolymerization of PPy on Au Wires

**FIGURE 3:**
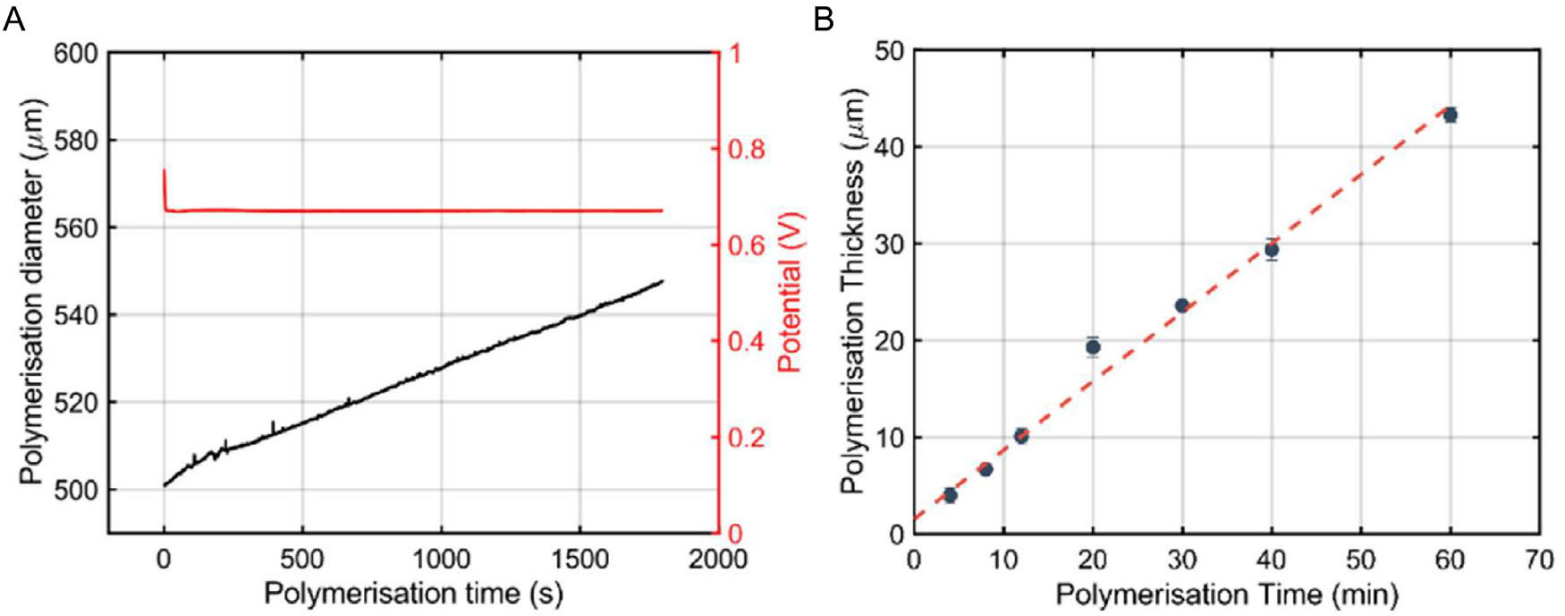
(A) Potential evolution (red) and noncontact optical measurements (black) of the deposition of PPy during a galvanostatic polymerization of PPy onto 500 μm diameter Au wires at + 0.3 mA cm^−1^ for 1800s (30 min) in 0.1 м Pyrrole, 0.1 м NaDBS aqueous solution. (B) PPy thickness obtained from 4, 8, 12, 20, 30, 40, and 60 min long galvanostatic polymerizations at + 0.3 mA cm^−1^. Standard deviation calculated from three independent experiments.

To fabricate the PPy coated Au wires, PPy was galvanostatically polymerized at + 0.3 mA per centimeter (cm) of Au wire immersed in the electrolyte, for 4, 8, 12, 20, 30, 40, and 60 min, in a 0.1 M pyrrole, 0.1 M NaDBS aqueous solution. Figure 3A shows the typical potential response of the polymerization of PPy on Au due to the applied current: it started with a short voltage peak during the first milliseconds that rapidly became constant at around + 0.7 V. Simultaneous noncontact optical measurements using the LSM showed an increase in the diameter of the wire, corresponding to an average PPy thickness growth rate of 0.74 ± 0.08 μm min^−1^. This rate was determined from polymerizations longer than 500 s, using the slope of the fitted curve beyond 500 s, since the deposition during the initial seconds was significantly faster. As expected, Figure 3B shows that increasing the polymerization time resulted in thicker polymer layers, also shown by Melling et al. for a similar PPy on Au system [52], and as indicated by the regression line in Figure 3B, red dashed line (x=0.71x+1.57 with *R* ^2^ = 0.988). The slope of this relationship closely matches the experimentally observed deposition rate. The higher slope during the first seconds could also have an influence on the ordinate at the origin being slightly different from 0.

#### 3.1.2 Electrochemical and Mechanical Characterization via CV

The electrochemical activity of the PPyAu wire actuators was confirmed by sweeping the potential between −1.0 and + 0.5 V at 10 mA s^−1^ in 0.1 м NaDBS aqueous solution for 15 cycles. Overall, the PPy coating exhibited good electrochemical activity, showing characteristics redox peaks, regardless of the polymerized thickness. However, during the first ten cycles, the redox peaks shifted slightly. From the 10th cycle onwards, the polypyrrole showed a stable reversible redox cycle with four clearly differentiated peaks: two reduction peaks and two oxidation peaks. Similar CV shapes were observed with similar synthesis and actuation conditions as well as with PPy doped with different dopants [63, 64, 65]. Figure S1 displays all fifteen cycles of the typical CV response of PPyAu wire actuator with 43 μm thick PPy. For this particular PPy thickness, the second to the fourth cycles presented only one reduction peak at −0.8 V and another oxidation peak at −0.2 V. From the 5th cycle to the 10th cycle, two extra peaks appeared upon both the reduction and oxidation of the polymer, at −0.35 and 0.1 V, respectively, and the response became stable from that point onwards.

**FIGURE 4:**
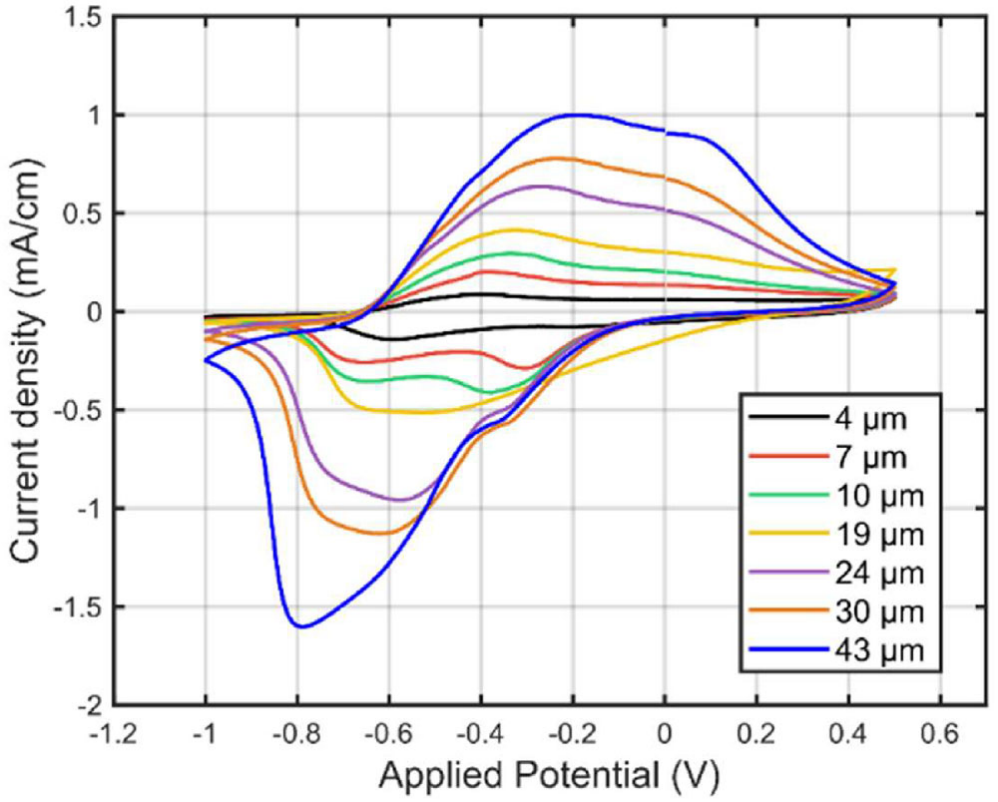
Current density response of the 15th cycle of PPyAu wire actuators with 4, 7, 10, 19, 24, 30, and 43 μm thick PPy when sweeping the potential between −1 and + 0.5 V at 10 mV s^−1^ 15 times in 0.1 м NaDBS aqueous solution.

The CV response changed with the thickness of the polypyrrole. Figure 4 shows the different current responses of polypyrrole layers deposited with different polymerization times, which resulted in different PPy thicknesses as mentioned previously. The more negative reduction peak gradually shifted from −0.6 to −0.8 V with increasing thicknesses whereas the first reduction peak stayed at around −0.3 V. The first oxidation peak moved from −0.4 to −0.2 V and the second one, more noticeable in thicker PPy layers, was first detected at around 0 V in the PPyAu wires with thinner PPy and slowly shifted to 0.1 V in PPyAu wires with thicker PPy. A similar response was observed for increasing thicknesses in freestanding PPy(DBS) films actuated in NaCl aqueous electrolytes [64]. Peaks started to separate as the layer of the polymer became thicker, indicating diffusion‐limited ion and solvent transport through the polymer film and time‐dependent internal polymer chain reorganization, which leads to delayed and depth‐dependent redox‐induced swelling and contraction (conformational relaxion) [66, 67, 68, 69, 70]. The current also increased with thicker layers of electropolymerized PPy, as predicted from the increasing charge consumption during the electropolymerization [71]. The charge consumption of the PPyAu wire actuators during the CV increased linearly with electrodeposited polymer thickness, ranging from an average of 7.5 mC cm^−1^ for the thinnest layers to 64.4 mC cm^−1^ for the thickest (Figure S2).

Simultaneous radial actuation measurements were conducted during the CV, which confirmed the radial expansion and contraction. Figure S3 shows the typical radial actuation obtained during the CV for the same PPyAu wire actuator with 43 μm thick PPy. Similar to the current and charge response, the radial actuation during the CV also increased with thicker polymer layers as shown in Figure S4. For the intended application of cellular mechanostimulation, precise control over stimulation amplitude, frequency, and duration is essential, as cellular responses are known to depend on both the magnitude and temporal characteristics of mechanical cues [4, 20, 72, 73, 74]. To achieve controlled and reproducible mechanical stimulation, radial actuation under square‐wave potential pulses is preferred to actuation under potential sweeps (such as CV). This is because square‐wave pulses provide easier control of pulse length and faster PPy volume changes in response to sudden redox potential switches. This actuation mode is examined in detail in the following section.

#### 3.1.3 Characterization of PPyAu Wire Actuators in NaDBS Aqueous Solution via Chronoamperometry

**FIGURE 5:**
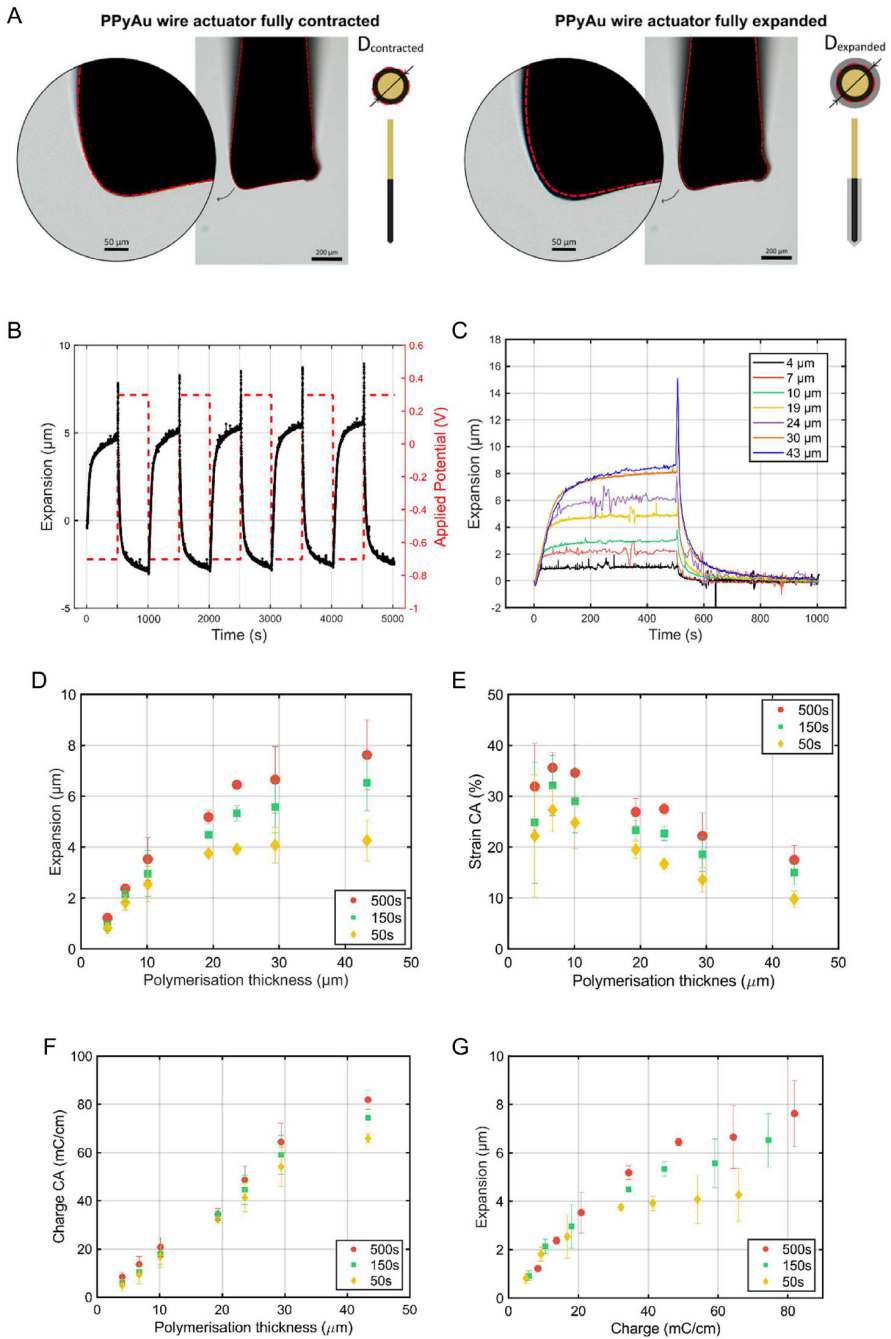
(A) Frames taken during the live stimulation of PPyAu wire when applying square wave potentials between −0.7 and + 0.3 V, with 150 s at each potential limit. The left image shows the PPyAu wire in its contracted state, while the right image shows it fully expanded. (B) The radial actuation of PPyAu wire actuator with 30 μm thick PPy measured using the LSM, (C) the charge consumption and (D) the current response during the application of square wave potentials ranging from −0.7 V to + 0.3 V, with 500 s at each potential limit. (E) The last cycle of the radial actuation measured using the LSM of PPyAu wire actuators with 4, 7, 10, 19, 24, 30, and 43 μm thick PPy. Average (D) radial actuation, (E) strain, and (F) charge for different PPy thicknesses during the application of square wave potentials ranging from −0.7 V to +0.3 V and (G) the average radial actuation versus the actuation consumed charge, with 50 (yellow), 150 (green), and 500 s (red) at each potential limit. Standard deviation calculated from three independent experiments.

After the electrosynthesis of the PPy and confirmation of its electrochemical activity using CV, the PPyAu wires were actuated in 0.1 м NaDBS aqueous solution by applying square‐wave potentials between −0.7 and + 0.3 V for 500 s at each potential limit. Since all PPyAu wires had already been preactuated during CVs and a stable response had been obtained, the actuation was prolonged for just five cycles, and all these five cycles were used to calculate the average expansion and contraction of the PPy layer. Figure 5A shows brightfield frames from the time‐lapse video of the actuation (Video S1), capturing the PPyAu wire actuator tip in its fully contracted (left) and fully expanded (right) states during the actuation. The red line outlines the perimeter of the fully contracted PPy and is overlaid on the expanded state to highlight the increase in PPy volume. These measurements were performed to enable brightfield, optical visualization of the actuator expansion, and to additionally corroborate the actuation behavior as measured using the LSM; the brightfield data therefore serve as complementary information to the LSM results presented next.

Figure 5B shows the typical radial actuation of a 30 μm thick PPyAu wire as measured with the LSM. Since the PPy was doped with big immobile DBS^−^ anions, the polymer showed the typical cation‐driven actuation [75]: PPy(DBS) expanded when Na^+^ ions entered the polymer upon reduction, i.e., applying −0.7 V, and contracted when the same Na^+^ ions exited the material during the oxidation, i.e., applying + 0.3 V. This is confirmed by X‐ray dispersion analysis in the following section. Figure S5A shows the charge and current curves of the PPy; the constant behavior of both actuation and charge suggests no signs of polymer degradation over time. However, it also reveals the characteristic asymmetric redox behavior of PPy(DBS), where the charge consumed during reduction is slightly greater (in absolute value) than that consumed upon oxidation [76, 77]. Figure S5B shows the current response during the actuation of the same PPyAu wire. As expected for a Faradaic system, each potential step exhibits a sharp current peak that decays exponentially towards zero within a few seconds. This consistent and repeatable current profile further indicates stable electrochemical performance with no observable polymer degradation throughout the cycling.

In Figure 5C, a single cycle of the expansion and contraction of the PPy is presented for each polymerization thickness. Regardless of the thickness of the PPy the radial actuation of the PPy occurs in two distinct phases. During the first seconds, the expansion or contraction is rapid and linear. In case of the thinnest PPy coatings, this is followed by a deceleration of the actuation until it reaches a plateau value. For the thicker PPy layers (20 μm or more), the second phase of the actuation remains linear but occurs at a slower rate. Melling et al. observed a similar behavior and attributed it to two different actuation phases [52]. According to the authors, and also Bay et al. [78], the initial phase is mainly driven by the movement of ions in or out of the polymer to compensate for the charge imbalance created by the sudden application of potential. During the second phase, the diffusion of the solvent to keep the osmotic pressure balance inside the polymer contributes to the actuation, a comparatively slower process.

The average radial actuation, strain, and charge after 50 s (yellow), 150 s (green), and 500 s (red) for each polymerization thickness are displayed in Figure 5D–F, respectively. The radial actuation was calculated as an average of the reversible expansion and contraction over five actuation cycles during the application of square‐wave potentials between −0.7 and + 0.3 V vs Ag/AgCl. Five cycles were sufficient since no difference was detected after the 10th cycle in previous CV studies (see previous section) and these PPyAu wires had already been pre‐actuated during CVs. In general, PPyAu wires with thicker PPy showed increasing radial actuation for 500 s long actuation pulses, up to a maximum of 7.6 ± 1.4 μm for 43 μm thick PPy layers. The radial actuation increased sharply with thickness in thinner PPy layers, but this trend began to plateau as the polymer became thicker. For short 50 s pulses, the maximum radial actuation reached a plateau value around 4 μm when the electropolymerized PPy was around 20 μm thick. Therefore, to achieve expansions of more than 4 μm thicker layers and/or longer time/slower cycles were needed.

This behavior is consistent with the diffusion‐based model described in Melling et al., and also similar models described in other papers but referred to as electrochemically stimulated relaxion model or diffusive‐elastic “metal” model, where the rate of ion and solvent transport into the PPy matrix and the rearrangement of the polymer matrix determines the extent of the actuation [52, 66, 67]. For short pulses, only the outer region is actively exchanging ions and solvent with the electrolyte and hence generating the actuation, while the inner regions remain inactive due to insufficient diffusion time. As the pulse duration increases, ions and solvent have the time to progressively penetrate deeper into the polymer, leading to increased actuation until a steady state is reached. At this point, the entire accessible portion of the PPy layer is engaged, and diffusion toward inner parts becomes so slow that it is not measurable. Thus, the actuation response is governed not only by polymerized thickness but also by the effective diffusion depth, which sets a practical limit on both the speed and amplitude of actuation.

Consequently, the optimal polymer thickness is application‐dependent and must be selected based on the desired balance between actuation amplitude, response time, and stimulation duration. Thinner PPy layers favor rapid and more homogeneous actuation, as the entire film can participate within short diffusion times. Thicker layers enable larger displacements under longer stimuli but may exhibit a faster expansion at the beginning of the actuation, and a slower actuation after some time, due to diffusion‐limited transport. In this sense, polymer thickness provides a tunable parameter to tailor the actuation profile to the requirements of a given mechanostimulation regime.

The calculated strain in Figure 5E shows a different behavior than the absolute radial actuation, the strain had a maximum of 35.6 ± 2.9% when the PPy was 6.7 ± 0.2 μm and thereafter decreased with thicker layers of PPy. A similar behavior was shown by Melling et al. [52] and Dutta et al. for linear yarn actuators [79]. The strain results further validate the diffusion‐based model. As the thickness increases the active PPy engaged in the actuation is relatively smaller compared to the total thickness of the polymer deposited during the electropolymerization, hence the reduction of the calculated strain.

As previously observed, the total charge consumed increased with polymer thickness and pulse duration (Figure 5F), but this increase plateaued as the polymer became thicker. The latter was particularly evident for short pulses (Figure 5G, yellow). This plateau confirms that beyond a certain thickness, the inner regions of the polymer become less accessible within the available diffusion time, limiting further charge exchange and actuation. Even at longer pulse durations (Figure 5G, red), the effect remained visible, reinforcing that the actuation in PPy is governed not just by total volume but by the effective diffusion depth.

Since all the PPy‐coated Au wires were previously characterized with a CV until stable cycling was achieved, the typical irreversible swelling characterized by Melling et al. and Smela et al. did not appear in the pulse actuation [51, 52]. However, these PPyAu wire actuators showed irreversible swelling when performing the CV as seen in Figure S3. Melling et al. studied and characterized the reversible and irreversible actuation of the PPy. Likewise, we saw that most of the irreversible swelling occurred in the first cycles, and the actuation became mostly reversible after the first 10–15 cycles, which coincided with the CV curves that became stable after around the same number of cycles.

We also observed spikes at the beginning of the potential cycles, but only in PPyAu wires with more than 10 µm thick PPy coatings. These spikes do not represent a sudden expansion of the polymer but were instead an effect of abrupt changes in local ion concentration, which momentarily change the refractive index near the polymer surface. Similar behavior was also reported by Melling et al. for thick polypyrrole films, and is therefore consistent with our observations [52].

#### 3.1.4 Characterization of PPyAu Wire Actuators in MEM

Since the mechanical stimulation of cells is performed in Minimal Essential Medium Eagle (MEM) cell medium inside an incubator, where the temperature is physiological (37°C), the actuation of PPyAu wires was also tested in this electrolyte at both 25 and 37°C to assess any actuation changes that the electrolyte or the temperature might induce in the actuation of the PPyAu wire. Cell medium is a complex electrolyte with several ions of different valency, Na^+^, K^+^, Ca^2+^, Mg^2+^, Cl^−^, CO_3_^2−^, SO_4_^2−^, and PO_4_^3−^ that can take part in the actuation of the PPy, as well as other elements such as proteins, and other biomolecules. Harjo et al. demonstrated the viability of the CP‐based actuators in cell medium but focused on the feasibility rather than a systematic comparison varying electrolytes and temperature [59]. Daneshvar and Smela showed that employing electrolytes that have a mixture of ions like those found on physiological fluids, as well as increasing the actuation temperature changed the electrochemistry of the polymer and the performance of the actuator. They found that changing from a simple electrolyte (NaDBS) to more complex medium (artificial cerebrospinal fluid, aCSF) slightly reduced the actuation, whereas increasing the temperature enhanced it in both NaDBS and aCSF [63]. Since all different cell media contain a different mixtures of ions and biomolecules at different concentrations, it is important to characterize the actuation of the PPy in such specific conditions.

Table S1 shows the concentration and the size of the solvated ions in MEM used here. For the characterization of the performance of PPyAu wires in MEM at room temperature and 37°C, they were polymerized with a fixed thickness of 20 μm. The electrochemical activity and the radial actuation of these wires were also previously confirmed in 0.1 м NaDBS aqueous solution to ensure reproducibility and leave them in comparable starting conditions. Thereafter, the NaDBS electrolyte was replaced with cell medium, and the temperature was increased.

**FIGURE 6:**
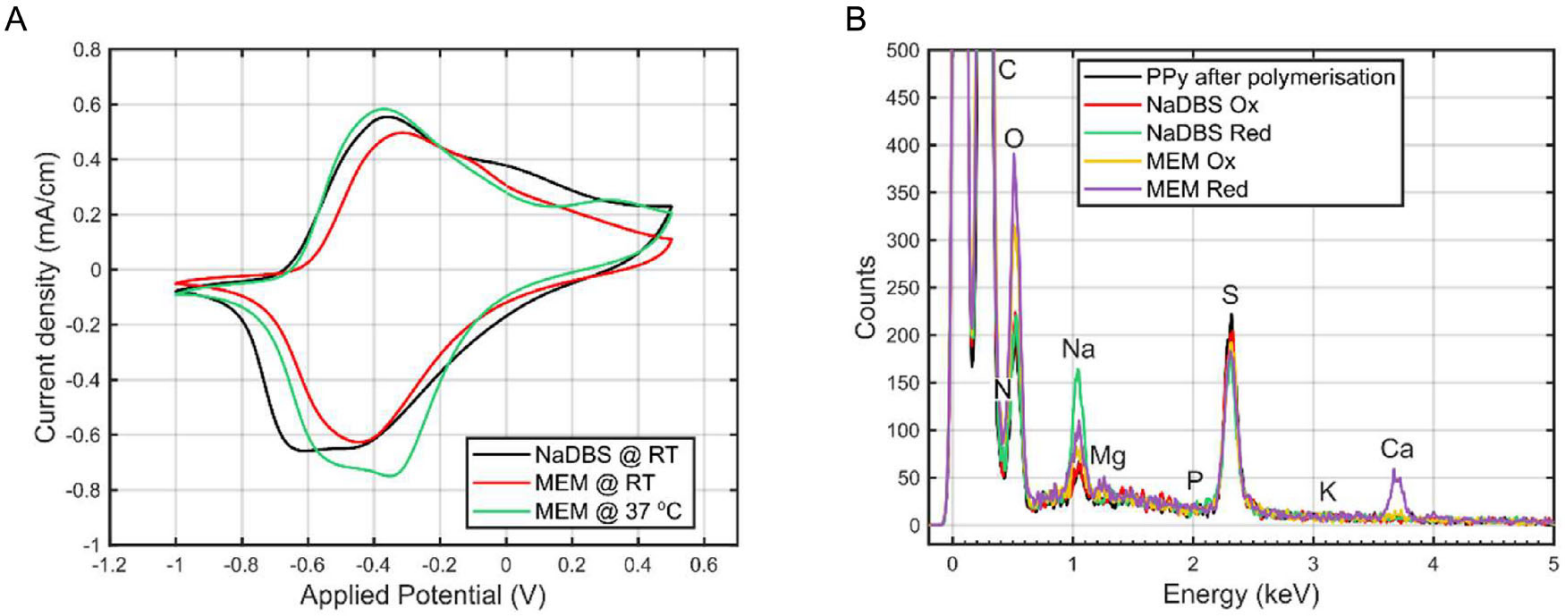
(A) Current denisty response of the last cycle of PPyAu wire actuators galvanostatically polymerized with a fixed thickness of 20 μm when ramping the potential between −1 and + 0.5 V at 10 mV s^−1^ 15 times in 0.1 м NaDBS aqueous solution (black) and MEM cell media (red). (B) Energy‐dispersive X‐ray spectroscopy (EDX) analysis of freshly polymerized polypyrrole doped with DBS^‐^ (PPyDBS), and after ten actuation cycles between −0.7 and + 0.3 V (150 s per potential limit) in either NaDBS or MEM electrolyte. Samples were analyzed in both the oxidized (contracted) and reduced (expanded) states. Peaks corresponding to O, Na, Mg, S, K, and Ca are highlighted.

Figure 6 shows the current responses during the 15th cycle of the CV, performed between [−1.0, +0.5] V at 10 mV s^−1^, for PPyAu wires with a 20 μm thick PPy in 0.1 м NaDBS at room temperature (black), MEM at room temperature (red) and MEM at 37°C (green). The PPyAu wire actuators showed very similar electrochemical response in cell media compared to the response in NaDBS, with reversible electrochemical cycles with clearly distinguished reduction and oxidation peaks. In 0.1 м NaDBS, the electrochemical response was defined by two reduction peaks at −0.6 V and a shoulder at −0.45 V, and well‐defined oxidation peak at −0.4 V with a shoulder at 0.0 V. When the electrolyte was changed to MEM at room temperature, the peaks shifted slightly toward more positive potentials, with a main reduction peak appearing around −0.4 V and oxidation features at approximately −0.3 V and a shoulder at −0.2 V. This shift may be attributed to the difference in ionic composition and conductivity between the NaDBS and the MEM electrolytes. Increasing the temperature of MEM to 37°C further altered the peak positions: the double reduction peak was observed again although shifted to around −0.5 and −0.3 V, while the oxidation peaks appeared at −0.4 and + 0.3 V. This temperature‐induced shift likely reflects changes in ionic mobility and altered electrochemical kinetics at physiological temperature. Overall, the data indicated that PPyAu wire actuators retained their reversible redox behavior across different media and temperatures, although new peaks or slight shifts in their positions reflect the influence of electrolyte composition and thermal conditions on the electrochemical response of the polymer.

Next, the radial actuation of the PPyAu wires in MEM at room temperature and physiological 37°C was investigated and compared to their actuation in NaDBS. Table 1 summarizes the average radial expansion of PPyAu wires during CV (column 3) across different electrolytes and temperatures. In NaDBS at room temperature (RT), PPyAu wires with 20 μm thick PPy expanded an average of 5.6 ± 0.6 μm. In MEM at RT, the average expansion was reduced to 3.6 μm, corresponding to ≈ 64.3% of the performance in NaDBS. This reduction is likely due to the presence of different ionic species in cell culture media, which have dissimilar radii and ionic valence and thus affect the number of ions exchanged and their mobility within the polymer matrix. When the temperature was increased to 37°C, the actuation in MEM improved slightly to 4.5 ± 0.1 μm, yet it remained lower than in NaDBS. Similar results were reported by Daneshvar and Smela: In their studies, they observed an increase in the bending angle of the bilayer actuator when increasing the temperature in both NaDBS and physiological media but a decrease when switching from NaDBS to the latter. However, the decrease in the actuation observed by them corresponded to only 6% [63]. These differences could again be attributed to differences in the composition of the media.

**TABLE 1:** The average expansion of 20‐μm‐thick PPyAu wire actuators measured using the LSM during the application of sweeping the potential between −1 and + 0.5 V at 10 mV s^−1^ 15 times and square wave potentials ranging from −0.7 V to + 0.3 V, with 150 s at each potential limit in 0.1 м NaDBS (black), in MEM cell medium and MEM cell medium at 37°C.

Electrolyte	Temperature	Cyclic voltammetry			Pulse actuation		
		Expansion, μm	Strain, %	Charge, mC cm^−1^	Expansion, μm	Strain, %	Charge, mC cm^−1^
NaDBS	RT	5.6 ± 0.6	28.4 ± 3.2	37.2 ± 3.3	6.0. ± 0.9	29.4 + 4.0	28.1 ± 1.1
MEM	RT	3.6 ± 0.2	18.0 ± 1.0	36.1 ± 1.1	3.1 ± 0.2	15.7 ± 0.6	24.6 ± 1.2
MEM	37°C	4.5 ± 0.1	22.1 ± 1.3	37.2 ± 9.3	3.1 ± 0.5	15.6 ± 2.8	22.4 ± 1.0

*Note:* Standard deviation is calculated from three different experiments.

Figure S6A presents the characteristic radial actuation in NaDBS at room temperature compared to MEM at room temperature and 37°C upon square wave potential stimulation ranging between [−0.7, +0.3] V for 150 s at each potential limit measured using the LSM. Again, the PPyAu wires showed a similar behavior when actuated in cell medium at both temperatures: a quick actuation during the first minute, during which most of the actuation was obtained, and a slower part during the remaining seconds, in which the actuation reached a slower yet steady actuation. PPyAu wires showed a radial expansion of 6.0 ± 0.9 μm in NaDBS. In contrast, in MEM both at RT and 37°C, the expansion was limited to 3.1 ± 0.3 μm, which represents 52.1% of the maximum actuation observed in NaDBS. This is in contrast to the actuation during CV and previous studies, in which higher temperatures enhanced the actuation in cell medium [63].

In addition to the mechanical performance, we also analyzed the charge consumed during the actuation. Table 1 shows the average charge consumed during both CV (column 5) and square wave pulses (column 8) in NaDBS (RT), MEM (RT), and MEM (37°C) and Figure S6B displays the charge obtained simultaneously with the potentiostat during the application of square wave potentials. During CV, the charge consumption was similar across all media and temperatures. However, during square wave pulsing, a slightly lower charge was consumed in MEM at both RT and 37°C compared to NaDBS, 87.5% and 79.6% of the charge, respectively. This may be attributed to the reduced participation of the wide variety of ions present in MEM within the narrower potential window used for pulsing [80]. This finding contrasts with the more significant differences observed in mechanical expansion. While the charge consumption remains relatively comparable, the mechanical output is notably reduced in MEM. This indicates that the actuation system is less performant in MEM, with a larger portion of the electrochemical work not being effectively converted into mechanical motion. Again, similar actuation efficiency results were reported by Daneshvar and Smela. They observed a substantial increase in charge consumption with rising the temperature, which, however, was not accompanied by a corresponding increase in the actuation.

In order to further clarify this, Figure 6B presents the elemental composition determined by X‐ray dispersion analysis for five different PPyAu samples: freshly polymerized PPy doped with DBS^−^; PPyDBS actuated in NaDBS for ten cycles between −0.7 and + 0.3 V for 150 s at each potential limit and left either in the oxidized (contracted) or reduced (expanded) state; and PPyDBS actuated in MEM for ten cycles between −0.7 and + 0.3 V for 150 s at each potential limit and also left in both redox states. Individual spectra can be found in the Supporting Information (Figure S7).

All samples showed signals for sodium (Na), carbon (C), sulfur (S), oxygen (O), and nitrogen (N). C is present on both the PPy backbone and the DBS dopant, N solely can be attributed to PPy, while S and O originate from DBS^−^, and Na from the NaDBS salt. The spectra of freshly polymerized PPy(DBS) and the sample actuated and left oxidized in NaDBS appeared remarkably similar, as would be expected. Upon reduction in NaDBS, the PPy(DBS) sample showed an increase in Na content, while the levels of C, N, S, and O remained consistent with those seen in both the oxidized and freshly polymerized states. This increase in Na is expected, as DBS^‐^ is a large, immobile anion embedded in the polymer matrix, and the only mobile charge‐compensating ion available during reduction is Na^+^. The resulting expansion of the polymer is thus driven by the insertion of Na^+^ ions, a well‐documented behavior for this system [57, 75, 81, 82], and is also consistent with the observed actuation: expansion during reduction (by insertion of Na^+^) and contraction during oxidation (by expulsion of Na^+^).

In contrast, the PPy(DBS) sample actuated in MEM and left in the oxidized state displayed higher levels of both O and Na compared to the oxidized sample in NaDBS. No additional anions were detected, which could be explained by the contracted state of the polymer; upon oxidation, positive charges are expelled from the polymer backbone. The reduced PPy(DBS) sample actuated in MEM revealed a reduced Na peak and a clear calcium (Ca) peak, which was absent in all other samples. A smaller increase in magnesium (Mg) was also observed. Despite potassium (K) being present in MEM at a higher concentration than both Ca and Mg (see Table S1), no significant increase in K signal was detected, nor there was a rise in phosphorus (P). This suggests that the swelling of PPy(DBS) in MEM is primarily driven by the uptake of monovalent Na^+^ and divalent Ca^2+^ ions, with a small exchange of Mg^2+^. The decrease of the Na peak together with the appearance of Ca and Mg suggest that part of the charge previously compensated by Na^+^ is now balanced by Ca^2+^ and Mg^2+^. The incorporation of these divalent cations likely contributes more significantly to the volumetric expansion of the polymer than monovalent ions such as K^+^, whose peak was not detected during the EDX analysis. This could also explain the lower expansion in MEM, as for every two electrons consumed now only one divalent ion is inserted instead of two monovalent Na^+^ ions. Additionally, both the reduced and oxidized samples cycled in MEM exhibited an increased oxygen signal. One possible explanation is the presence of more water molecules associated with the inserted Ca^2+^ ions as their hydration shell is larger, as seen in Table S1. This could result in a higher overall oxygen content detected in the EDX analysis. However, the larger hydration shell did not compensate for the lower number of inserted ions, and therefore the overall actuation remained smaller.

To evaluate the longer‐term performance of the actuator and to assess its behavior under different stimulation protocols, we performed extended actuation tests using both repeated short pulses and prolonged (30 min) stimulation. This is particularly relevant for future applications where various mechanostimulation protocols may be explored. Figure S8 shows the radial expansion of PPyAu wires actuated over 2 h, either (A) by repeating the same 150 s long pulses 72 times or (B) by prolonging the pulse for 30 min at each potential limit, in both NaDBS and MEM electrolytes at RT. The short‐cycle experiment (150 s pulses) demonstrated a consistent actuation response across all 72 cycles in both media. Notably, transient spikes were observed in NaDBS but not in MEM. As discussed earlier, these spikes are not due to actual polymer expansion but rather to abrupt changes in local ion concentrations, which alter the refractive index near the polymer surface.

Under prolonged (30 min) pulses, differences between the two electrolytes emerged. In NaDBS, the expansion reached a clear plateau, indicating that the polymer did not continue to expand under constant potential, suggesting saturation of ionic uptake, as it corresponds to a diffusion‐controlled process, as discussed before. The actuation in MEM exhibited redox and/or actuation asymmetry, with the polymer not achieving the same contraction upon oxidation as it expanded during reduction as seen by the upwards trend (Figure S8B). This incomplete switching may limit the actuation range and should be considered when designing mechanostimulation protocols, as well as be investigated further to determine how it can be avoided.

#### 3.1.5 Characterization of PPyAu Wire Actuators in Two‐ and Three‐Electrode Systems

To enable miniaturization and compatibility with standard well plates, we aim to simplify the electrochemical setup by reducing the number of electrodes. However, in two electrode systems, the potential drop between the CE and the electrolyte can result in a reduced effective voltage at the WE, leading to attenuated actuation of the polymer. This motivates the studies on both two‐ and three‐electrode configurations effects on the electrochemistry and the actuation of the PPyAu wire, which we quantify in the following section. Then, we attempted to counteract the actuation loss by applying a wider potential window.

**FIGURE 7:**
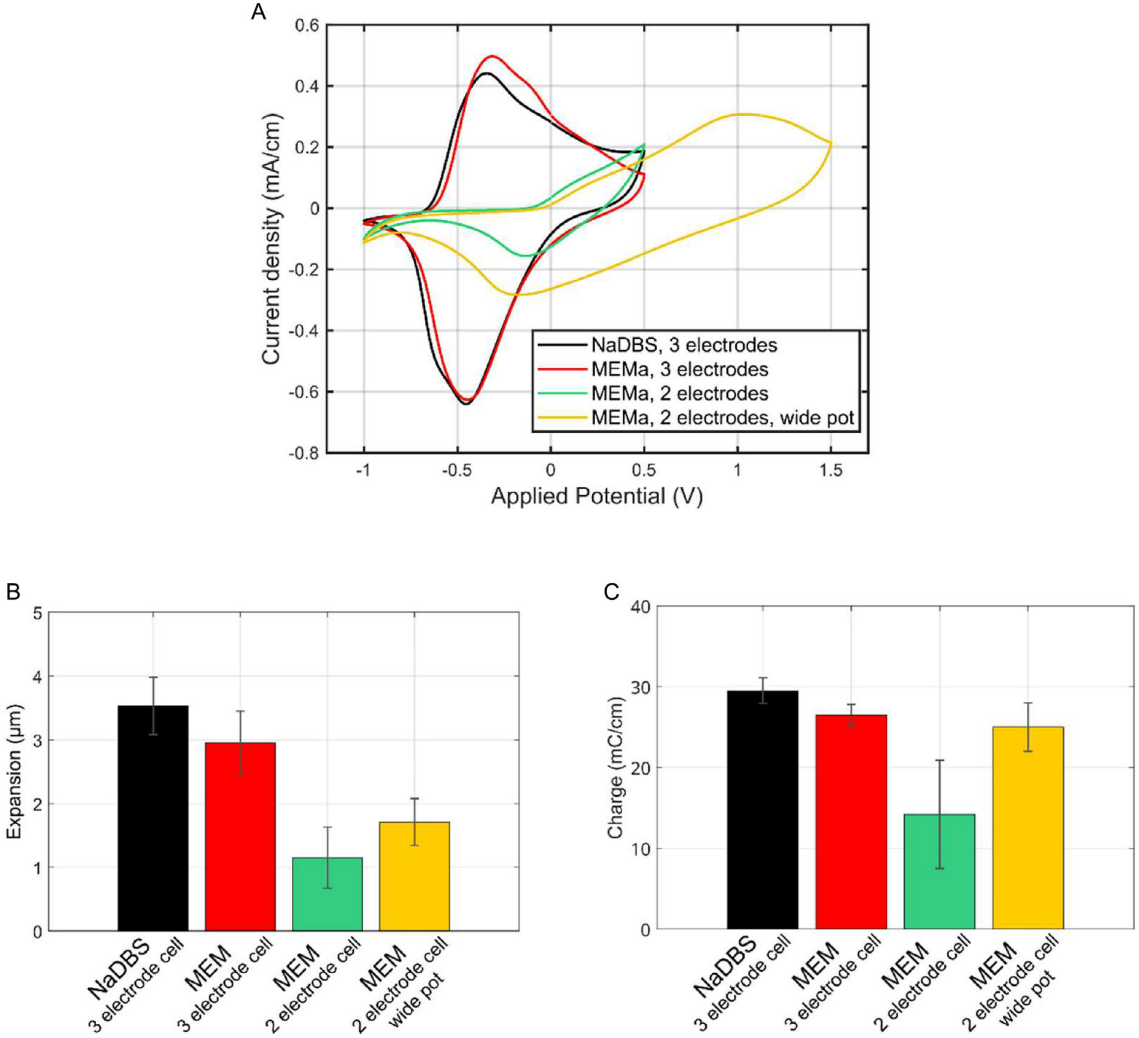
(A) Current density response of the last cycle of PPyAu wire actuators with a 19 μm thick PPy coating when ramping the potential between –1.0 and 0.5 V at 10 mV s^−1^ in 0.1  м NaDBS aqueous solution and MEM cell media using a three‐electrode configuration; in MEM cell media using a two‐electrode configuration; and between –1.0 and 1.5 V in a two‐electrode configuration in MEM cell media. (B) Radial actuation, and (C) charge of PPyAu wire actuators with a 19 μm thick PPy coating applying 500 s long square wave potentials −0.7 and + 0.3 V in 0.1 M NaDBS aqueous solution and MEM cell media using a three‐electrode configuration; in MEM cell media using a two‐electrode configuration; and between –1.0 and 1.5 V in a two‐electrode configuration in MEM cell media.

Figure 7A presents the CVs of PPyAu wires with a 19 μm thick PPy layer when cycled between −1.0 and + 0.5 V at 10 mV s^−1^. Measurements were performed in a three‐electrode system using NaDBS and MEM electrolytes, and in a two‐electrode system using MEM. Additionally, a two‐electrode configuration was evaluated with an extended potential window between −1.0 and + 1.5 V in MEM.

In the two‐electrode configuration, a clear shift of the reduction peak toward more positive potentials was observed compared to the three‐electrode system. Notably, the oxidation peak was no longer visible within the standard potential window (from −1.0 to + 0.5 V), suggesting a similar positive shift. When the potential range was extended to + 1.5 V, the oxidation peak could be observed around + 1 V, confirming this hypothesis.

As seen in the bar charts in Figure 7B, using a two‐electrode configuration whilst applying the standard potential window significantly reduced the radial actuation in cell media. Despite applying a wider potential window, the magnitude of the actuation did not match that achieved in the three‐electrode system. As displayed in Figure 7C, the total charge consumed in the two‐electrode setup with the standard potential window remained lower than in the three‐electrode configuration too, indicating incomplete redox cycling of the polymer. However, when using the extended potential window, the charge consumption increased significantly, though this was not accompanied by greater expansion. This suggests that part of the charge may have been consumed by other side reactions occurring at the counter electrode (CE) or the Au beneath the PPy on the WE.

These results indicate that although a wider potential window can partially compensate for the loss of charge and actuation in a two‐electrode configuration, it does not fully restore the actuator's achievable strain amplitude. In particular, the lower charge consumption and diminished radial actuation demonstrate that full and reversible redox cycling of the PPy is not achieved in the two‐electrode setup. The use of a three‐electrode configuration is therefore preferred to ensure complete and efficient redox cycling and optimal actuation.

### 3.2 Mechanical Stimulation of KUSA‐A1 Osteoblasts

**FIGURE 8:**
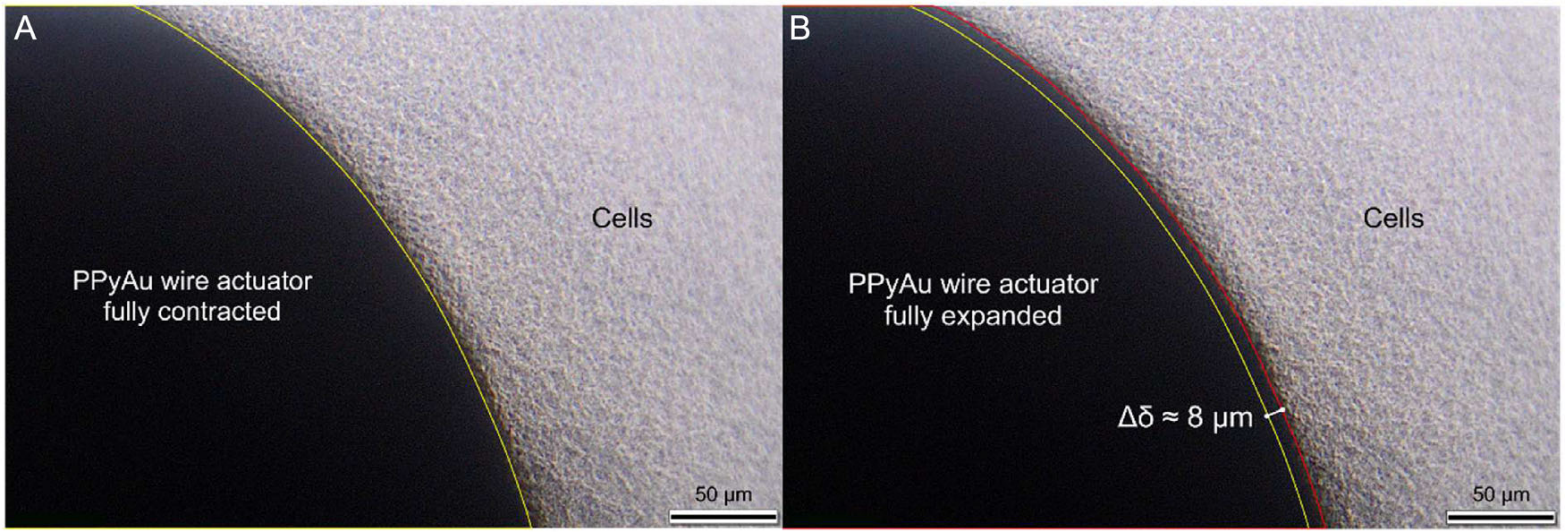
Bright field image of the PPyAu wire actuator fully (A) contracted and (B) expanded and in direct contact with osteoblasts.

To explore the potential of PPyAu wires as a mechanotransduction tool, we here demonstrate mechanical stimulation of KUSA‐A1 osteoblastic cells. These cells are widely used for studies of bone metabolism and mechanotransduction [19], making them suitable for demonstrating proof‐of‐concept cellular mechanostimulation using the proposed actuator. For cellular stimulation, we employed PPyAu wires with a PPy thickness of 20 µm, which provided micrometre‐scale radial expansion (∼4 µm), comparable to the characteristic dimensions of osteoblastic cells (∼20‐50 µm lateral size, ∼2‐5 µm thick) [83], within the initial phase of actuation (first ∼ 50 s under our experimental conditions). To simulate the mechanical forces in an in vitro model, we adapted the electrochemical setup to fit within a 24‐well plate and replaced the Ag/AgCl RE with the Ag wire pseudo RE. After seeding the osteoblasts onto the membrane of the inserts and allowing them to attach to the membrane, the PPyAu wire actuator was inserted perpendicularly into the membrane, piercing both the confluent cell layer and the membrane. The cells remained in direct contact with the PPyAu wire actuator, extending around its surface, which ensured a high degree of cell‐wire actuator interface throughout the stimulation. Figure 8 presents bright field images extracted from a time‐lapse video recorded during the stimulation of osteoblasts (Video S2). The video was recorded to demonstrate that the actuator operates effectively in the presence of cells and to directly visualize cell displacement induced by actuation, replicating the configuration used in the incubator‐based experiments. On image A, the PPyAu wire actuator is fully contracted, whereas on image B, the wire actuator is fully expanded, exerting mechanical force on the surrounding osteoblasts positioned along the perimeter of the actuator. The yellow semicircle outlining the diameter of the actuator is the same size in both images. The red semicircle in image B outlines the expanded diameter of the actuator. The difference between the two diameters depicts the expansion of the PPy adjacent to the cell. Based on scale, that difference is calculated to be around 8 μm, which is remarkably larger than the radial expansion measured with the laser scanner during the characterization, yet still within the expected range. This confirms not only that the actuator functions in presence of the cells, but also that the induced mechanical displacement is effectively transmitted to the cells, resulting in observable changes in their position.

The stimulation of the osteoblasts was performed using 18 cycles of 100‐second pulses between −0.7 and + 0.3 V inside the incubator (37°C and 5% CO_2_), which resulted in a total mechanical stimulus of 30 min. Since in situ optical measurements were not feasible inside the incubator, the actuation of the wires was verified using the LSM prior to the cells’ mechanical stimulation. Charge consumption and current profiles during the mechanical stimulation in vitro were then compared to preactuation data to confirm correct PPy actuation (Figure S9). As shown in Figure 5G, the volume generally increases proportionally with charge transfer and therefore, current and charge measurements serve as good performance indicators when live actuation recordings are not feasible. Directly after the stimulation, cells located in the proximity of the wire were collected for further RNA analysis.

Some mechanotransduction pathways are activated by the application of current to cells [62]. However, the aim of this work is to isolate and study the effect of mechanical stimulation alone. To ensure that any observed effects were not due to the surface properties nor the electrical stimulation, two control experiments were conducted. In the first control, the same stimulation protocol was applied using an uncoated Au wire. This wire also perforated the confluent cell layer and membrane, i.e., was positioned in close proximity to the cells, mirroring the setup used with the PPyAu wire actuator. As shown in Figure S10A, the charge consumption recorded for the bare Au wire (green) was significantly lower than that of the PPyAu wire actuator (red), as the resulting current was minimal (Figure S10B). This indicates that the uncoated Au wire is not the optimal control for separating the effect of the ionic current. Therefore, a second control was included. In this experiment, a fully coated PPyAu wire was placed in the cell medium but positioned just above the cell layer, without making direct contact with the cells. Since a smaller portion of the active polymer layer was immersed, the charge was normalized per unit length of the active part, i.e., immersed in the medium. This wire underwent radial expansion and contraction similarly to the ones used for mechanical stimulation, but without physically affecting the cells. Given the same PPy coating and thickness, the electric field and ionic current in the medium were comparable to those in the mechanical stimulation experiments (Figure S10A,B, yellow vs. red), allowing for a more accurate assessment of these effects.

Mechanical loading and unloading of osteoblasts have previously been shown to alter gene expression [84]. Therefore, RNA gene expression analysis can provide insights into the cellular changes induced by mechanical stress, as well as the downstream effects of such stimulation. However, important questions regarding the optimal duration, frequency, and magnitude of the mechanical cues, and whether cells respond differently depending on the specific characteristics of the stimulus remain unclear. The PPyAu wire actuators have the potential to provide mechanical stimulation for several hours and different pulse lengths while keeping the cells inside the incubator. Here, we illustrate how the PPyAu wire actuator can be used to elucidate such questions as a short proof of concept. In particular, we aim to exemplify that we can induce gene expression alteration with our method. Table 2 shows the upregulated (orange) and downregulated genes (blue) from all pair comparisons between the stimulation and the control groups.

**TABLE 2:** Upregulated (orange) and downregulated (blue) genes upon pair comparison between groups.

Pair comparison	Biotype	Subcategory	Gene name/ensemble gene ID
Stimulation vs Control 1	lncRNA	Uncategorized	Unknown/ENSMUSG00000135935
Stimulation vs Control 2	Protein coding	Gene regulation	Fos
			Fosb
	lncRNA	Uncategorized	Gm47939
Stimulation vs Control 3	lncRNA	Uncategorized	Gm45027
Control 1 vs Control 2	lncRNA	Uncategorized	2 310 014F06Rik
Control 1 vs Control 3	lncRNA	Uncategorized	D530017H19Rik
Control 2 vs Control 3	Protein coding	Signaling	Dmp1
			Hrc
			Lss
			Fyn
			Deptor
			Enpp2
			Cxcl12
			Tgfbr3
		ECM/Structure	P3h2
			Col11a2
			Col24a1
			Thbs2
			Fbn1
		Gene regulation	Fmr1
			Ahr
		Cell Adhesion/Morphology	Arvcf
			Plxna2
		Cell viability/Stress response	Gas6
		Others	9 930 111J21Rik2
	Pseudogene	Uncategorized	Gm15725
			Gm12435
			Gm9800
			Eif4a‐ps4
	MtRNA	Cellular metabolism	mt‐Rnr1
			mt‐Rnr2
	lncRNA	Uncategorized	Gm50445
			Snhg4
	SnoRNA	Uncategorized	Snora73b
			Snord3b4

The aim of comparing the PPyAu wire actuator stimulation with the Au wire was to isolate the mechanical effect from the electrical stimulation. PPyAu wires expanded and contracted stimulating the cells (*Stimulation*), and Au wires served as control since they do not undergo dimensional changes but allow current flow (*Control 2*). Notably, two genes, *Fos* and *Fosb*, were upregulated that are known to respond to stress and mechanical forces and play a role in bone remodeling [85]. In addition, the long noncoding RNA Gm47939 was found to be downregulated, although its function is unknown. Therefore, additional experiments and optimized protocols, such as longer stimulation times, are required to obtain a more comprehensive overview of the changes in RNA expression induced by mechanical stimulation. However, electrochemical analysis revealed that the current generated by applying a potential to the Au wire did not fully replicate the current produced during stimulation with PPyAu wires. Consequently, it cannot be conclusively determined that the observed changes in gene expression are solely attributable to mechanical stimulation in this case.

Comparing the PPyAu wire actuator without any applied potential (*Control 1*) and the Au wire with the applied potential (*Control 2*) captures transcriptional changes arising from small currents, the proximity of Au to cells, or the effect of PPy. RNA analysis identified one upregulated long noncoding RNA, 2 310 014F06Rik, whose function remains uncharacterized. Given that this was the only upregulated lncRNA detected and that it does not encode a protein, the results indicate that the PPyAu and Au wire with applied potential produce similar cellular responses and no damage to the cells.

In *Control 3*, the PPyAu wires were positioned without direct contact with the cells but generated a current comparable to that observed in the mechanically stimulated cells (*Stimulation*), as confirmed by simultaneous electrochemical measurements, see Figure S9. Notably, the comparison between the PPyAu wire actuator above the cells (*Control 3*) and the Au wire (*Control 2*) resulted in the largest number of gene expression changes, indicating that these alterations are likely driven by the current or electric field in the cell culture medium. However, neither *Fos* nor *Fosb* were upregulated in this comparison, suggesting that their upregulation in the *Stimulation* versus *Control 2* comparison is predominantly induced by mechanical stimulation of the PPyAu wire actuator rather than by the effect of the high currents. More experiments are also required to understand why genes upregulated under high versus low current conditions are not similarly upregulated when both mechanical and electrical stimulation are applied (*Stimulation*) compared with the remaining groups, despite comparable high currents being measured.

While the present study focuses on a single osteoblastic cell line as a proof‐of‐concept, the PPyAu wire actuator platform is not inherently cell‐type specific and is designed to be adaptable to other mechanically sensitive cell types. Ongoing in‐depth studies are aimed at evaluating its effectiveness across different osteoblastic models under varied stimulation conditions. In addition, the system has already been applied to HUVEC cultures and other cellular models, with further systems currently under investigation. These preliminary results indicated that mechanical stimulation of cells via PPyAu wire actuators is feasible and constitutes a powerful tool for advancing our understanding of cellular mechanotransduction. They also revealed a highly complex system that requires careful experimental design and interpretation to ensure that the mechanostimulation experiments and control groups yield meaningful insights.

## 4 Conclusions

In this work, we developed and characterized a simple, easy‐to‐use, PPyAu wire actuator dedicated for mechanical stimulation of cells in standard incubator environments and demonstrated its utility by mechanically stimulating KUSA‐A1 osteoblasts.

The main part of this work focused on developing the method, i.e., investigating the fabrication and characterization of PPy(DBS) on a Au wire using a noncontact optical method. An increased galvanostatic polymerization time resulted in thicker PPy, as also shown by Melling et al. [52]. Electrochemical and actuation characterization showed that the expansion behavior of PPy(DBS) films varied with thickness and pulse length. Thicker films (>20 µm) exhibited limited expansion under short pulses (50 s), reaching a plateau around 4 µm expansion. Longer pulses were required to achieve larger expansions up to of 7.6 ± 1.4 μm for 43 μm thick PPy, allowing sufficient time for ion diffusion. The strain had a maximum of 35.6 ± 2.9% for 6.7 ± 0.2 μm thick PPy. We then examined the electrochemical and actuation behavior in cell medium, at room temperature and 37°C. Differences in the ionic content in the actuation electrolyte led to different ion insertion, as confirmed by EDX analysis, and decreased actuation in cell medium from 6.0 ± 0.9 μm to 3.1 ± 0.2 μm. Lastly, two‐electrode systems revealed reduced actuation at similar charge consumption, likely due to shifted oxidation potentials and undesired side reactions, pointing to the need of using a three‐electrode system for accurate experiments.

In the last part, the system was implemented in commercial well‐plates to mechanically stimulate in vitro KUSA‐A1 osteoblastic cell line cultured on insert membranes inside the incubator. The electrochemical behavior of the PPyAu wire actuators during stimulation aligned well with prior characterization results. Osteoblasts that were mechanically stimulated showed upregulation of *Fos* and *Fosb*, both involved in mechanosensitive gene regulation and bone remodeling. Moreover, several differentially expressed genes remain uncharacterized, hinting at the involvement of previously unknown components in the mechanotransduction pathway. Current control experiments have identified genes that are upregulated in response to the electric field and/or ionic current, which provides us with a list of genes that are transcribed due to the current and not the mechanical stimulation, and should be considered in the design of future experiments.

Beyond this proof of concept, the PPyAu wire actuator offers considerable flexibility: although we here demonstrated a single, straight wire/needle actuator, the wire can be shaped into different geometries (e.g., Z‐shaped actuators for otherwise inaccessible regions) or combined into multiple individually controlled actuators arranged in lines or arrays to produce more complex stimulation patterns. While we have demonstrated in vitro use, the same concept could be extended to ex vivo or even in vivo applications.

Beyond the technical advancement, this work lays the groundwork for future biological studies by presenting a new mechanostimulation tool that can deliver diverse mechanical cues within standard culture conditions and different cell microenvironments. Using this tool in mechanobiology could help unravel how cells respond to different mechanical inputs, which can shed light on various unknown mechanotransduction pathways. Ultimately, this will advance our knowledge of the effects of mechanical forces at the cellular level and how the latter can influence the onset and progression of diseases such as thrombosis, cancer, or osteoporosis.

## Supporting Information

Additional supporting information can be found online in the Supporting Information section. **Supporting Figure S1**: Current density response of PPyAu wire actuator with 43 μm thick PPy (60 min of polymerization) when ramping the potential between ‐1 and +0.5 V at 10 mV/s 15 times in 0.1 M NaDBS aqueous solution. **Supporting Figure S2**: Average charge for different PPy thicknesses when sweeping the potential between ‐1 and +0.5 V (red) at 10 mV/s for 15 cycles in 0.1 M NaDBS aqueous solution. Standard deviation calculated from three independent experiments. **Supporting Figure S3**: The radial actuation (black) of PPyAu wire actuator with 43 μm thick PPy (polymerized for 60 min at +0.3 mA/cm) when sweeping the potential between ‐1 and +0.5 V (red) at 10 mV/s for 15 cycles in 0.1 M NaDBS aqueous solution. **Supporting Figure S4**: Average radial actuation for different PPy thicknesses when sweeping the potential between ‐1 and +0.5 V (red) at 10 mV/s for 15 cycles in 0.1 M NaDBS aqueous solution. Standard deviation calculated from three independent experiments. **Supporting Figure S5**: The radial actuation of 20 μm‐thick PPyAu wire actuators (A), measured using the LSM, and the charge consumption (B) during the application of square wave potentials ranging from −0.7 V to +0.3 V, with 150 s at each potential limit in 0.1 M NaDBS (black), in MEM cell medium (red) and MEM cell medium at 37°C (green). **Supporting Figure S5**: The radial actuation measured with the LSM, (C) the charge consumption and (D) the current response during the application of square wave potentials ranging from −0.7 V to +0.3 V, with 500 s at each potential limit, of PPyAu wire actuator with 30 μm thick PPy. **Supporting Figure S6**: The radial actuation of 20 μm‐thick PPyAu wire actuators (A), measured using the LSM, and the charge consumption (B) during the application of square wave potentials ranging from −0.7 V to +0.3 V, with 150 s at each potential limit in 0.1 M NaDBS (black), in MEM cell medium (red) and MEM cell medium at 37°C (green). **Supporting Figure S7**: Individual energy‐dispersive X‐ray spectroscopy (EDX) analyses of freshly polymerized polypyrrole doped with DBS_−_ (PPyDBS) (black), and after 10 actuation cycles between −0.7 and +0.3 V (150 s per potential limit) in either NaDBS in the oxidized (red) and reduced (green) states or in MEM electrolyte in the oxidized (yellow) and reduced (purple) states. Peaks corresponding to O, Na, Mg, S, K, and Ca are highlighted. **Supporting Figure S8**: Radial expansion of PPyAu wires actuated over 2 hours using repeated 150 s pulses (A) or prolonged 30 min pulses (B) in NaDBS and MEM electrolytes at room temperature. **Supporting Figure S9**: Radial expansion (A), charge consumption (mC/cm) (B), current density (mA/cm) (C) of the PPyAu wire when being pre actuated in 0.1 M NaDBS between [‐0.7, +0.3] V for 150 s at each potential limit compared to the charge consumption (mC/cm) (D) and current (mA/cm) (E) of the same PPyAu wire when performing the mechanical stimulation of cells inside the incubator. **Supporting Figure S10**: Charge (mC/cm) (A) and current (mA/cm) (B) responses of PPyAu wire actuation during the mechanical stimulation of osteoblasts (red), Au wire inserted in the membrane next to the cells (green) and PPyAu wire actuating slightly above the cells. Applied potential: square wave potentials between ‐0.7 and +0.3 V for 100 s at each potential limit. **Supporting Table S1**: Ionic content of Minimal Essential Medium Eagle (mM) and their solvated radii (pm).

## Author Contributions

H.K. and E.W.H.J. conceived and supervised the project. A.B.O.S. designed, performed, and analyzed the electrochemical experiments with input from J.G.M. and E.W.H.J., and together with S.H. designed, performed, and analyzed the cell mechanostimulation experiments with input from S.H., J.G.M., H.K., and E.W.H.J. S.H. and E.S.H. carried out the RNA sequencing and subsequent data analysis. A.B.O.S. wrote the manuscript and generated the figures and illustrations with input from all other authors.

## Funding

This work was supported by the Japan Society for the Promotion of Science (JP23KK0163, JPJSBP120209923); Swedish Foundation for International Cooperation in Research and Higher Education (MG2019−8171); Vetenskapsrådet (VR2019−0368); Linköpings Universitet (SFO‐Mat‐LiU No. 2009 00971).

## Conflicts of Interest

The authors declare no conflicts of interest.

## Supporting information

Supplementary Material

## Data Availability

The data that support the findings of this study are available from the corresponding author upon reasonable request.
