# Supplementary material for: PPy‐Coated Wire Actuators for the Micromechanostimulation of Cells: Fabrication and Characterization
Source: Small Sci. 2026 Mar 13;6(3):e202500639. doi: 10.1002/smsc.202500639 (PMC13097529; doi:10.1002/smsc.202500639)
Supplement: Supplementary file 1 — Supplementary Material [file SMSC-6-e202500639-s001.zip › New folder/SI Small s.1.pdf]

Supporting Information

PPy-coated Wire Actuators for the Micromechanostimulation of Cells – Fabrication and Characterisation

Amaia B. Ortega-Santos[1], Satoru Hayano[2], Emilio Satoshi Hara[3], Jose G. Martinez[1], Hiroshi Kamioka[4], Edwin W. H. Jager[1]

[1] *Department of Physics, Chemistry and Biology (IFM), Linköping University, Linköping, Sweden.*

[2] *Department of Orthodontics, Okayama University Hospital, Okayama, Japan.*

[3] *Advanced Research Center for Oral and Craniofacial Sciences Dental School, Okayama University Graduate School of Medicine, Dentistry and Pharmaceutical Sciences, Okayama, Japan.*

[4] *Department of Orthodontics, Okayama University Graduate School of Medicine, Dentistry and Pharmaceutical Sciences, Okayama, Japan.*

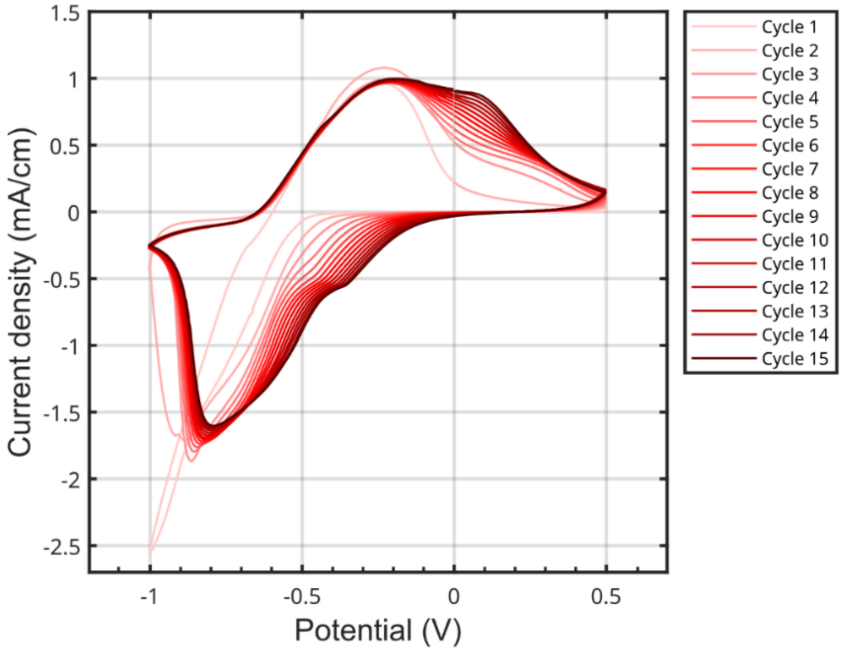

Supporting Figure 1 Current denisty response of PPyAu wire actuator with 43 µm thick PPy (60 min of polymerisation) when ramping the potential between -1 and +0.5 V at 10 mV/s 15 times in 0.1 M NaDBS aqueous solution.

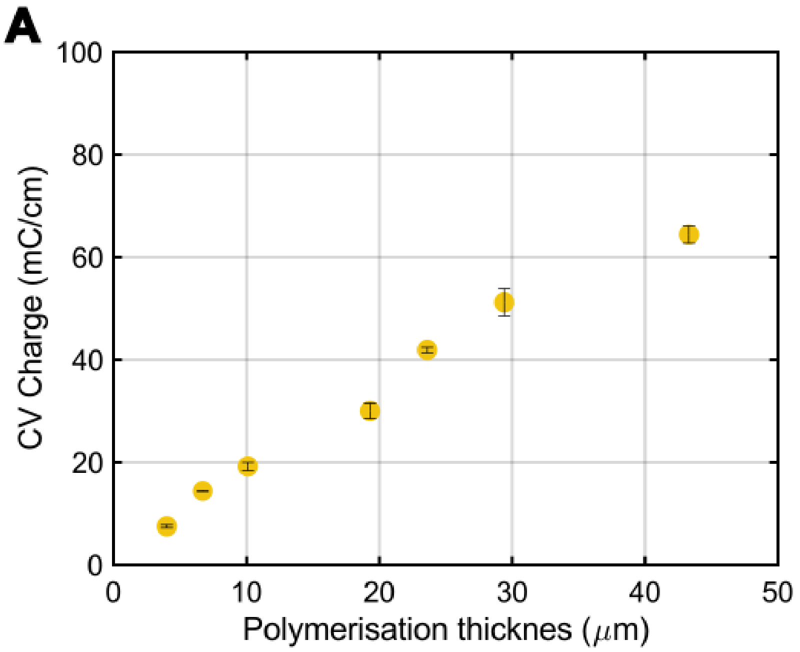

Supporting Figure 2 Average charge for different PPy thicknesses when sweeping the potential between -1 and +0.5 V (red) at 10 mV/s for 15 cycles in 0.1 M NaDBS aqueous solution. Standard deviation calculated from three independent experiments.

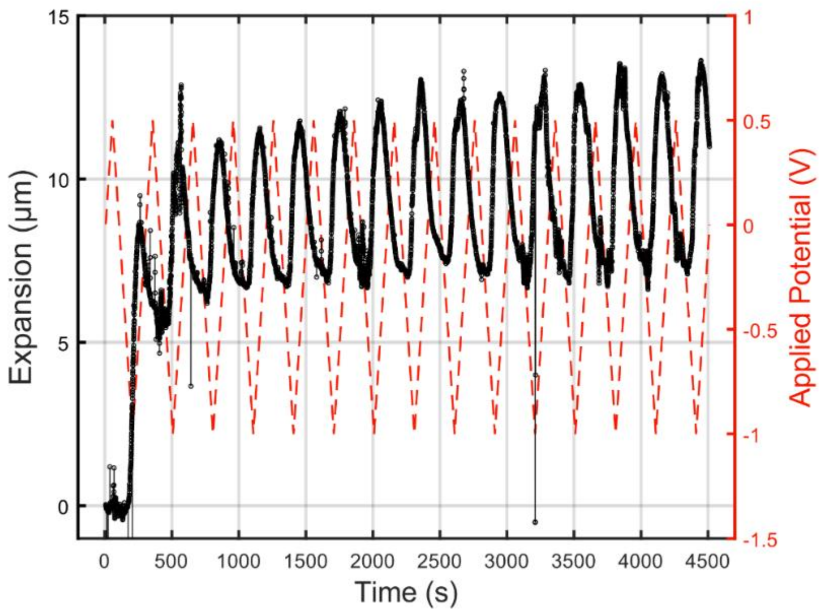

Supporting Figure 3 The radial actuation (black) of PPyAu wire actuator with 43 µm thick PPy (polymerised for 60 min at +0.3 mA/cm) when sweeping the potential between -1 and +0.5 V (red) at 10 mV/s for 15 cycles in 0.1 M NaDBS aqueous solution

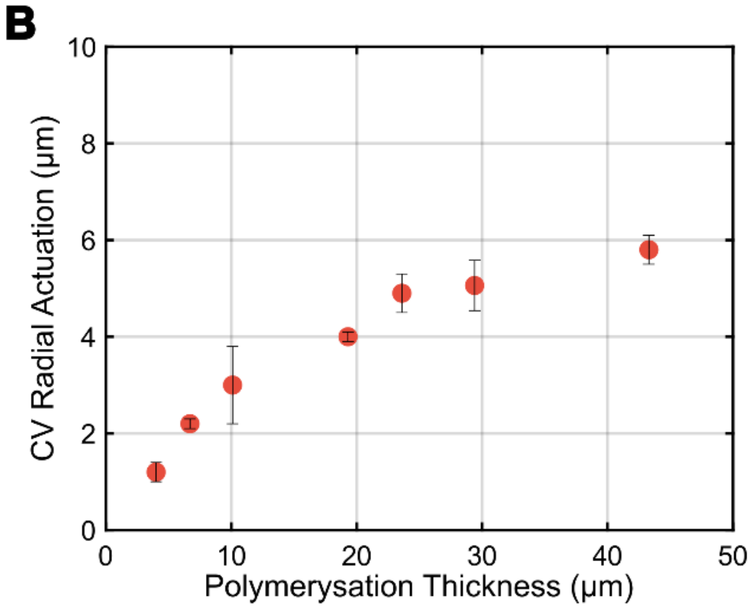

Supporting Figure 4 Average radial actuation for different PPy thicknesses when sweeping the potential between -1 and +0.5 V (red) at 10 mV/s for 15 cycles in 0.1 M NaDBS aqueous solution. Standard deviation calculated from three independent experiments.

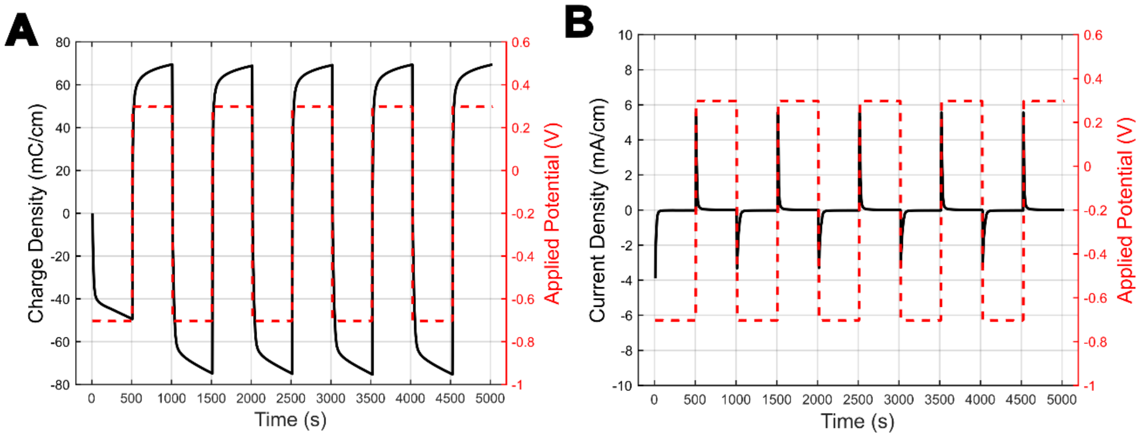

Supporting Figure 5 The radial actuation of 20 µm-thick PPyAu wire actuators (A), measured using the LSM, and the charge consumption (B) during the application of square wave potentials ranging from −0.7 V to +0.3 V, with 150 s at each potential limit in 0.1 M NaDBS (black), in MEM cell media (red) and MEM cell media at 37 ºC (green).

Supporting Figure 5 The radial actuation measured with the LSM, (C) the charge consumption and (D) the current response during the application of square wave potentials ranging from −0.7 V to +0.3 V, with 500 s at each potential limit, of PPyAu wire actuator with 30 µm thick PPy.

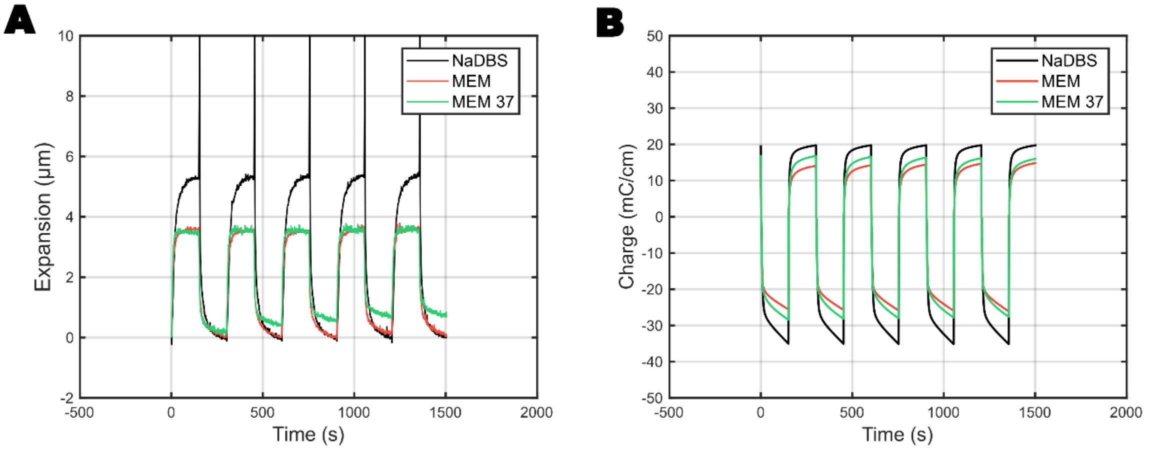

Supporting Figure 6 The radial actuation of 20 µm-thick PPyAu wire actuators (A), measured using the LSM, and the charge consumption (B) during the application of square wave potentials ranging from −0.7 V to +0.3 V, with 150 s at each potential limit in 0.1 M NaDBS (black), in MEM cell media (red) and MEM cell media at 37 ºC (green).

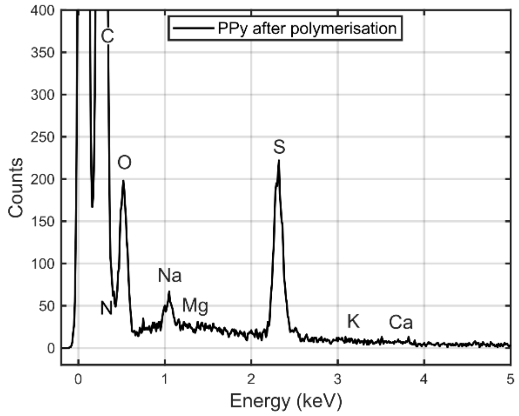

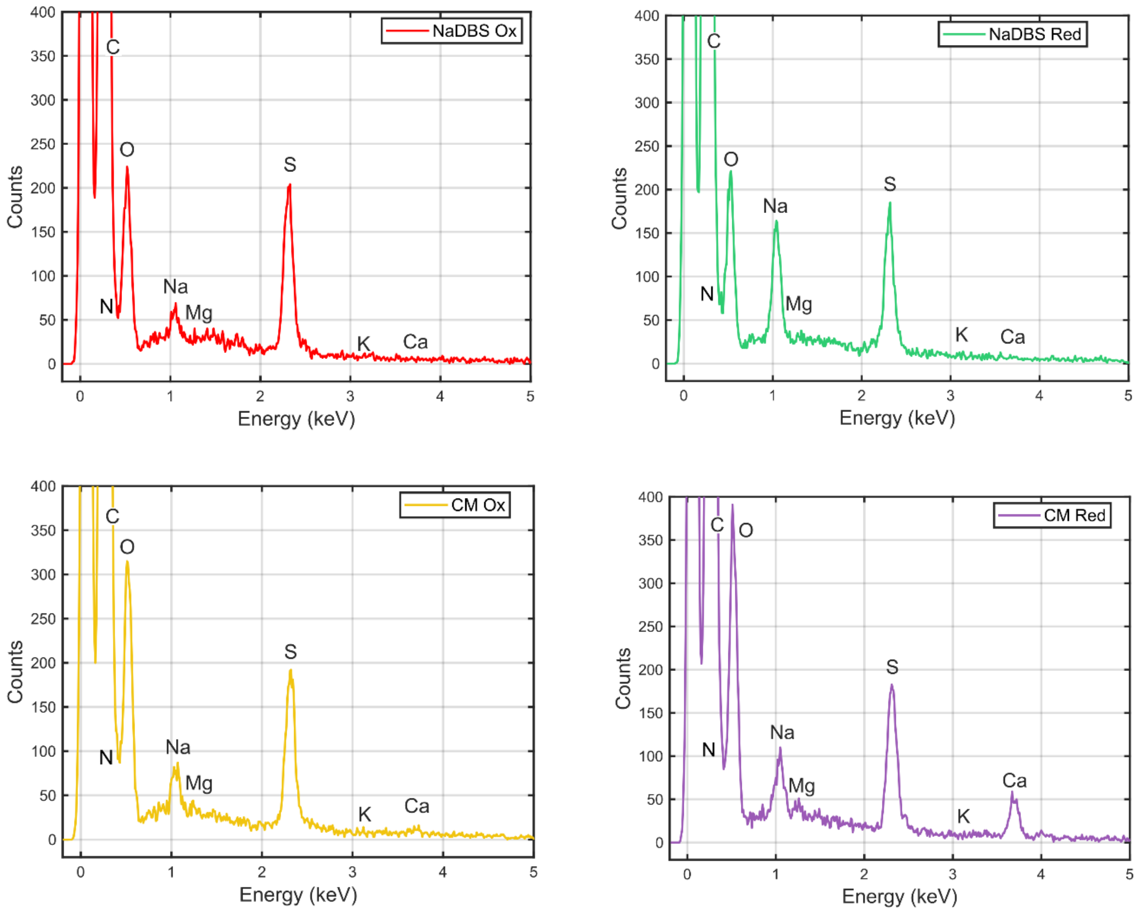

Supporting Figure 7 Individual energy-dispersive X-ray spectroscopies (EDX) analysis of freshly polymerised polypyrrole doped with DBS⁻ (PPyDBS) (black), and after 10 actuation cycles between −0.7 and +0.3 V (150 s per potential limit) in either NaDBS in the oxidised (red) and reduced (green) states or in MEM electrolyte in the oxidised (yellow) and reduced (purple) states. Peaks corresponding to O, Na, Mg, S, K, and Ca are highlighted.

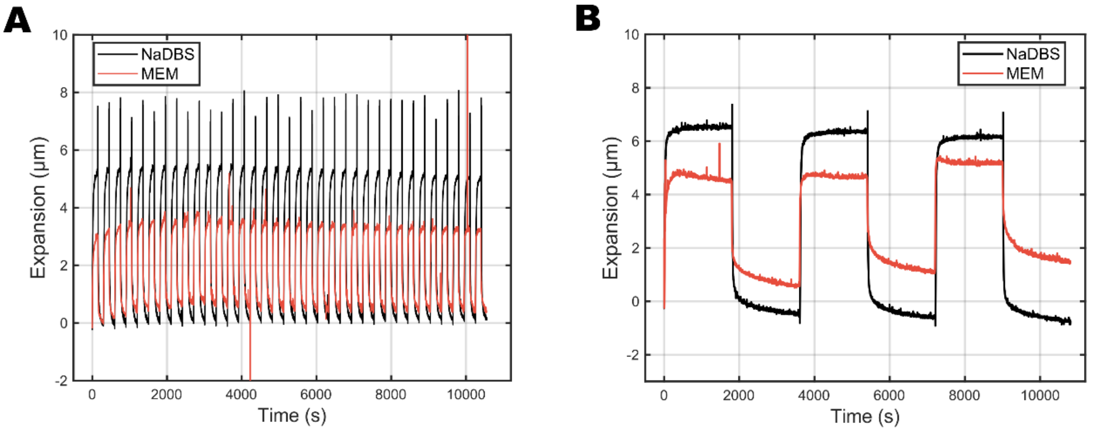

Supporting Figure 8 Radial expansion of PPyAu wires actuated over 2 hours using repeated 150 s pulses (A) or prolonged 30 min pulses (B) in NaDBS and MEM electrolytes at room temperature.

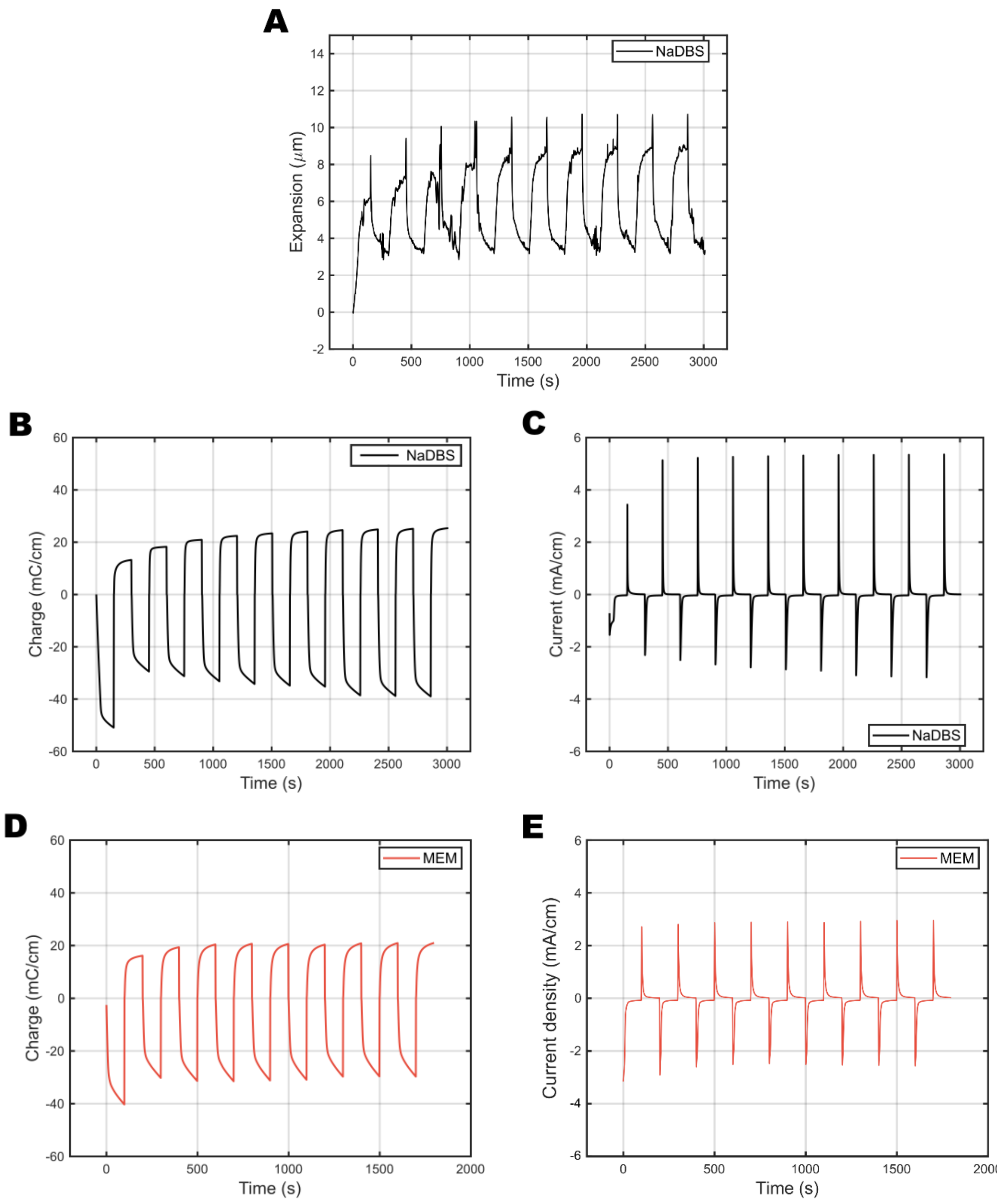

Supporting Figure 9 Radial expansion (A), charge consumption (mC/cm) (B), current density (mA/cm) (C) of the PPyAu wire when being pre actuated in 0.1 M NaDBS between [-0.7, +0.3] V for 150 s at each potential limit compared to the charge consumption (mC/cm) (D) and current (mA/cm) (E) of the same PPyAu wire when performing the mechanical stimulation of cells inside the incubator.

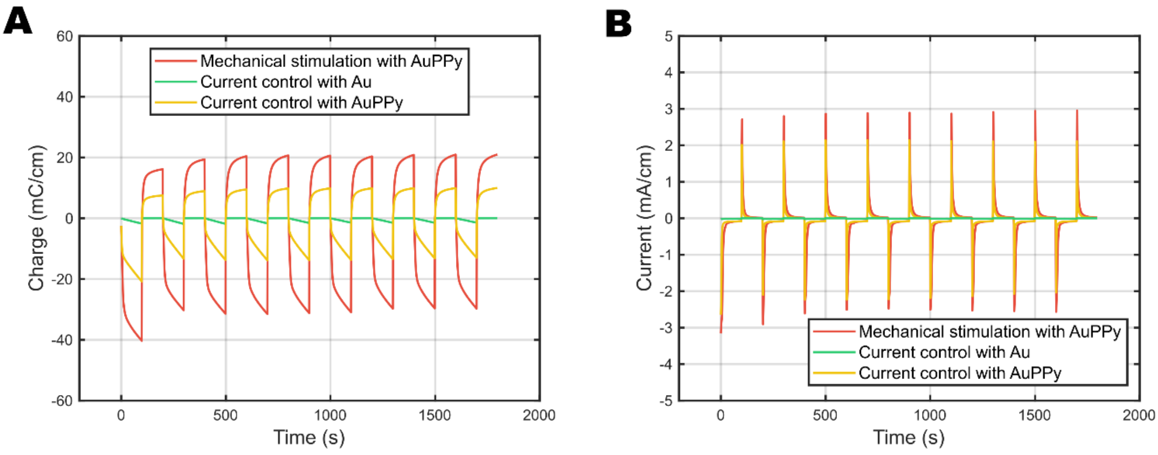

Supporting Figure 10 Charge (mC/cm) (A) and current (mA/cm) (B) responses of PPyAu wire actuation during the mechanical stimulation of osteoblasts (red), Au wire inserted in the membrane next to the cells (green) and PPyAu wire actuating slightly above the cells. Applied potential: square wave potentials between -0.7 and +0.3 V for 100 s at each potential limit.

**Ion**	**Solvated radii (pm)**	**Concentration (mM)**	**Primary Source**
Na^+	450	143.6	NaCl, $NaHCO_3$, NaH_2PO_4
Cl^-	300	125.4	NaCl, KCl, $CaCl_2$
K^+	300	5.36	KCl
Ca^{2+}	600	1.8	$CaCl_2$
Mg^{2+}	800	0.81	$MgSO_4$
HCO_3^-	450	26.2	$NaHCO_3$
HPO_4^-	400	1.0	$NaH_2PO_4 \cdot H_2O$
SO_4^{2-}	400	0.81	$MgSO_4$

Supporting Table 1 Ionic content of Minimal Essential Medium Eagle (mM) and their solvated radii (pm).
